# Molecular and Cellular Interactions in Pathogenesis of Sporadic Parkinson Disease

**DOI:** 10.3390/ijms232113043

**Published:** 2022-10-27

**Authors:** Lyudmila P. Dolgacheva, Valery P. Zinchenko, Nikolay V. Goncharov

**Affiliations:** 1 Federal Research Center “Pushchino Scientific Center for Biological Research of the Russian Academy of Sciences”, Institute of Cell Biophysics of the Russian Academy of Sciences, Pushchino 142290, Russia; 2 Research Institute of Hygiene, Occupational Pathology and Human Ecology of the Federal Medical Biological Agency, Saint Petersburg 188663, Russia; 3 Sechenov Institute of Evolutionary Physiology and Biochemistry of the Russian Academy of Sciences, Saint Petersburg 194223, Russia

**Keywords:** Parkinson’s disease, α-synuclein, dopamine, Lewy bodies, reactive oxygen species, calcium ions, substantia nigra, gut microbiota, endothelial cells, organophosphates, nutraceuticals

## Abstract

An increasing number of the population all around the world suffer from age-associated neurodegenerative diseases including Parkinson’s disease (PD). This disorder presents different signs of genetic, epigenetic and environmental origin, and molecular, cellular and intracellular dysfunction. At the molecular level, α-synuclein (αSyn) was identified as the principal molecule constituting the Lewy bodies (LB). The gut microbiota participates in the pathogenesis of PD and may contribute to the loss of dopaminergic neurons through mitochondrial dysfunction. The most important pathogenetic link is an imbalance of Ca^2+^ ions, which is associated with redox imbalance in the cells and increased generation of reactive oxygen species (ROS). In this review, genetic, epigenetic and environmental factors that cause these disorders and their cause-and-effect relationships are considered. As a constituent of environmental factors, the example of organophosphates (OPs) is also reviewed. The role of endothelial damage in the pathogenesis of PD is discussed, and a ‘triple hit hypothesis’ is proposed as a modification of Braak’s dual hit one. In the absence of effective therapies for neurodegenerative diseases, more and more evidence is emerging about the positive impact of nutritional structure and healthy lifestyle on the state of blood vessels and the risk of developing these diseases.

## 1. Introduction

Parkinson’s disease (PD) is the second most common neurodegenerative disorder affecting approximately 1% of the population over the age of 50. PD is clinically characterized by uncontrollable tremors at rest, rigidity, slowness of movement and postural impairment. In addition to violations of motor function, PD is accompanied by gastrointestinal, olfactory, sleep, and cognitive pathologies and other disorders. PD is characterized by a progressive loss of dopaminergic neurons in the substantia nigra pars compacta (SNpc) [1]. These neurons release dopamine (DA) from nerve endings in the striatum and control muscle tone. DA neurons of the SNpc are controlled by GABAergic neurons from another SN region (SN pars reticulata). With the fall in striatal DA levels below 70–80%, the clinical manifestations of PD, including bradykinesia, resting tremor, rigidity and postural instability develop [2,3]. Morphologically PD is characterized by the presence of intracellular inclusions called Lewy bodies (LB) consisting mainly of aggregated α-synuclein (αSyn) [4,5] inside nerve cells including SNpc.

The key features of PD are the accumulation of aggregated αSyn, mitochondrial disorders, neuroinflammation, and the disruption of the blood–brain barrier (BBB) function [6]. All of these factors are involved in damage to dopaminergic neurons. Dysfunction of the mitochondria (MCh) plays a critical role in both sporadic and hereditary forms of PD and is one of the hallmarks of PD [7,8]. Mitochondrial disorders are among the earliest biochemical signs of the disease and are manifested in disturbances in the interaction of MCh and the endoplasmic reticulum (ER), calcium homeostasis, and an increase in the level of reactive oxygen species (ROS). Mitochondrial dynamics, including division, fusion, and movement within the cell, are an important function of DA neurons since the axons of neurons of the nigrostriatal system constitute one of the longest tracts in the brain and are associated with certain difficulties in the delivery of ATP to transport components to the distally located synaptic terminals [9]. In the pathogenesis of PD, there are several kinds of disturbances in bioenergetics in the cells of the nigrostriatal system: a decrease in the activity of complex I of the mitochondrial respiratory chain, an increase in the production of ROS by MCh, the ROS-mediated damage to mitochondrial DNA and of other molecules, disturbed mitophagy, followed by various functional disorders of MCh and their host cells. Elevated levels of ROS and energy deficit disrupt the interactions between MCh and ER, reduce the function of lysosomes, and disrupt calcium homeostasis [10]. Oxidative stress factors promote αSyn aggregation [11]. Aggregated αSyn accumulates within the neuronal cell in the nigrostriatum, and is deposited in LB, which first appears in the olfactory bulb, the intestinal or enteric nervous system (ENS), and the medulla oblongata. Under pathological conditions, misfolded αSyn is secreted from donor cells as a naked protein or in vesicles to be further transported to other cells [12,13,14,15]. This is facilitated by the expression of inflammatory cytokines that disrupt the blood–brain barrier (BBB) [16]. Other factors than inflammatory cytokines, e.g., cell membrane receptors, such as low-density lipoprotein receptor-related protein 1 (LRP1), lymphocyte activation gene 3 (LAG3) and amyloid precursor-like protein 1 (APLP1) are involved in cell-to-cell transmission [17,18].

## 2. Familial and Sporadic Forms of PD Epigenetic Aspects

The onset of PD is dependent on both genetic and environmental factors. The latter can alter gene expression by causing epigenetic changes, such as DNA methylation, and the post-translational modification of histones and non-coding RNAs (ncRNAs, the most studied of which are microRNAs or miRNAs). The regulation of genes responsible for monogenic forms of PD may also be involved in sporadic PD [19].

### 2.1. Genetic Factors

Mutations of seven genes have been definitely linked to PD, and these are PARK1/4 (SNCA, encoding α-synuclein), PARK2 (PRKN, encoding parkin), PARK7 (encoding parkinsonism associated deglycase, best known as DJ-1), PINK1 (encoding phosphatase and tensin homolog (PTEN)-induced kinase 1), LRRK2 (encoding leucine-rich repeat kinase 2), VPS35 (encoding vacuolar protein sorting ortholog 35, involved in autophagy), and GBA1 (GTP-binding protein type A1, encoding lysosomal β-glucocerebrosidase 1) [20,21,22,23,24,25,26]. Mutations in three genes, PARK1/4 (SNCA), LRRK2 and VPS35, are known to cause a dominant form of PD, whereas mutations in PARK2 (PRKN), PINK1, and PARK7 cause recessive-inherited forms of the disease [27]. Mutations in GBA1 are causal for the rare autosomal storage disorder Gaucher disease, and they are also among the most commonly known genetic risk factors for the development of Parkinson’s disease and related synucleinopathies. A least 495 different mutations, found throughout the 11 exons of the gene are reported, which may lead to the degradation of the protein, disruptions in lysosomal targeting and diminished performance of the enzyme in the lysosome [26]. As for those indefinitely linked to PD, more than 100 genes or genetic loci have been identified, and most cases likely arise from interactions among many common and rare genetic variants [28]. 

### 2.2. Environmental Factors

Nevertheless, most cases (85–90%) of PD are believed to be sporadic. Generally, sporadic PD is a progressive neurodegenerative disease, which is characterized by signs and symptoms of progressive motor and non-motor dysfunctions (e.g., sleep disturbance) [29]. The sporadic forms of PD are associated with various aggressive environmental factors, including the effects of neurotoxins (e.g., 1-methyl-4-phenyl-1,2,3,6-tetrahydropyridine, MPTP; its derivative 1-methyl-4-phenylpyridinium, or MPP+, interferes with the complex I), pesticides and herbicides, such as rotenone and paraquat [30,31,32], as well as factors, such as aging, traumatic brain injury, vascular risk factors, hypertension, diabetes mellitus, obesity and metabolic syndrome, depression, drugs addiction, physical inactivity, smoking, and alcohol consumption [33,34,35,36]. Such impacts lead to the appearance of an excess of electrons, the formation of ROS, and oxidative stress. These processes are most intensive in dopaminergic neurons of SN, which eventually die and no longer control the striatal neurons that maintain muscle tone.

**Figure 1:**
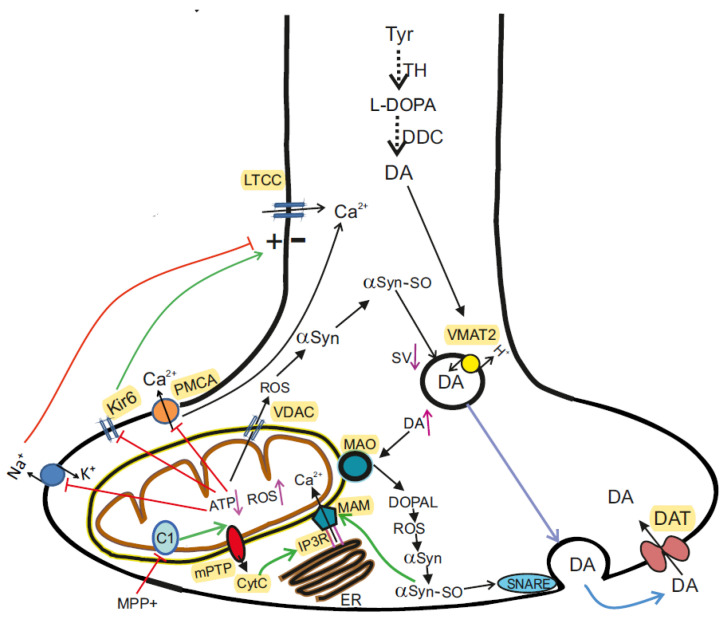
Neurotoxins initiate sporadic PD. The primary motor symptoms of PD are caused by a progressive decrease in neuronal DA in the striatum. The required level of DA is provided by its synthesis and reuptake and can be recycled for exocytosis with the help of DA transporter in the presynapse. Inhibition of mitochondrial complex I (C1) by neurotoxin (MPP+) leads to decreased ATP synthesis and generation of ROS, followed by inhibition of plasma membrane ATPases (Na+/K+ATPase, PMCA), opening of Kir6, cell hyperpolarization and decreased activity. With a lack of ATP, the activity of H^+^-ATPase coupled with VMAT2 decreases and DA levels rise in the cytosol. Excess DA can be metabolized by MAO-B to the toxic metabolite DOPAL, which promotes oxidative stress, mPTP opening, and death of dopaminergic neurons. Under conditions of excess ROS or Ca^2+^, αSyn undergoes aggregation. The aggregated αSyn (αSyn-SO) breaks the process of SNARE complex assembling and clustering, docking and fusion of DA-filled synaptic vesicles. Aggregated αSyn leads to functional impairment of the MAM complex, mPTP opening, release from MCh of cytC, which interacts with the IP3 receptors and keeps the Ca^2+^ channels open. Abbreviations: αSyn—α-Synuclein; DATs—dopamine transporters; DOPAL—3,4-dihydroxyphenylacetaldehyde; Kir6—ATP-sensitive K^+^ channels; LTCCs—L-type calcium channels, a long-opening high-voltage-gated calcium channels; MAO—monoamine oxidase; MAM—mitochondria-associated membranes; PMCA—plasma membrane Ca^2+^-ATPase; VMAT2—vesicular monoamine transporter 2.

The molecular pathogenesis of sporadic forms includes not only oxidative stress, but also many other pathways and mechanisms: mitochondrial dysfunction, αSyn proteostasis, calcium homeostasis, axonal transport, and neuroinflammation. Mitochondrial dysfunction plays a fundamental and complex role in many neurodegenerative disorders, including PD [37]. PD-associated mitochondrial dysfunction can result from a number of causes, including impairment of mitochondrial biogenesis, increased ROS production, defective mitophagy, compromised trafficking, electron transport chain (ETC) dysfunction (Figure 1), variations to mitochondrial dynamics, calcium imbalance and possibly other indirect influences on mitochondrial function from unrelated pathways [38,39]. 

The involvement of MCh in the pathogenesis of PD was first identified following human consumption of illicit drugs contaminated with MPTP. Symptoms resembling those of PD were observed shortly after taking the drugs, and pathoanatomical studies revealed the destruction of SN [40]. Subsequent studies have shown that MPTP is oxidized by monoamine oxidase B (MAO-B) to its toxic bioactive form MPP+ which enters DA-producing neurons in the SN via the DA reuptake system [41]. When entering the cell, MPP+ inhibits the mitochondrial NADH-ubiquinone oxidoreductase of the ETC Complex I (Figure 1) and leads to electron leakage and the formation of ROS in MCh [42].

Braak and colleagues postulated a hypothesis that microbial pathogens (viruses or bacteria) in the gut could be responsible for the initiation of sporadic PD [43]. This was followed 4–6 years later by the dual-hit hypothesis of the same scientific team, according to which sporadic PD starts in two places: the neurons of the nasal cavity and the neurons in the gut, spreading via the olfactory tract and the vagal nerve, respectively, toward and within the central nervous system (CNS) [44,45]. Braak’s hypothesis is supported by in vitro, in vivo, and clinical evidence, though the staging system of Braak only describes a specific subset of patients with young onset and long duration of the disease [46]. 

Most cases of sporadic PD are characterized by abnormal accumulation and aggregation of αSyn within neuronal cells in the nigrostriatum, which is converted into amyloid fibrils and deposited in LB. Post-mortem brain studies of people ranging from early stages of PD to advanced stages show that LB first appears in the olfactory bulb, the gut nervous system, and the dorsal motor nucleus of the vagus located in the medulla oblongata [47,48]. LB then appears in the locus coeruleus of the pons, the raphe nucleus in the pontine midbrain, and SNpc in the midbrain. In the advanced stages of the disease, LB is found in the temporal cortex, limbic region, and cerebral cortex. For example, αSyn expression was enhanced in the nigrostriatum of the rotenone-induced Parkinson’s rats [49]. Thus, PD is characterized not only by the abnormal accumulations and aggregation of αSyn within neuronal cell bodies and neuritis but also by the intercellular transport of aggregated αSyn [12,13]. 

A study of the intestinal nervous system has shown that disturbances in intestinal permeability and systemic exposure to bacterial antigens induce the expression of inflammatory cytokines, such as tumor necrosis factor (TNF-α) or interleukin (IL)-1β and IL-6, which disrupt the integrity of the BBB, contribute to the accumulation of αSyn in SN and lead to the death of dopaminergic neurons [50].

### 2.3. Epigenetic Aspects in Development of PD

Epigenetics is the study of heritable changes in gene expression that occur without alterations to the DNA sequence, linking the genome to its surroundings. In recent years, a lot of evidence has emerged that genes associated with PD are particularly prone to epigenetic dysregulation. On the other hand, in a healthy young person, the accumulation of epigenetic alterations over the lifespan in genes not associated with PD may also contribute to neurodegeneration, and these epigenetic biomarkers may be useful in clinical practice for the diagnosis, surveillance, and prognosis of disease activity in patients with PD [51,52,53,54]. 

Some genes underlying PD loci would alter PD risk through changes to expression or splicing. Gene-level analysis of expression revealed five genes (WDR6 [OMIM 606031], CD38 [OMIM 107270], GPNMB [OMIM 604368], RAB29 [OMIM 603949], and TMEM163 [OMIM 618978]) that replicated. A further six genes (ZRANB3 [OMIM 615655], PCGF3 [OMIM 617543], NEK1 [OMIM 604588], NUPL2 [NCBI 11097], GALC [OMIM 606890], and CTSB [OMIM 116810]) showed evidence of disease-associated splicing effects [55]. Transcriptional regulation tightly correlates with specific epigenetic marks. In X-linked dystonia-parkinsonism, three disease-specific single-nucleotide changes (DSCs) introduce (DSC12) or abolish (DSC2 and DSC3) CpG dinucleotides and consequently sites of putative DNA methylation [56]. Current research indicates that variants in the *SNCA* gene, exposure to pesticides, and physical activity impact the epigenome, particularly at the level of CpG methylation, so these factors are key contributors to PD risk [57,58]. On the other hand, of the fourteen analyzed CpGs of SNCA_intron1_, CpGs 16–23 were hypomethylated in PD [59]. 

Elevated αSyn levels may influence the epigenetic regulation of PD pathways, too. In sporadic PD, the gastrointestinal tract may be a site of origin for αSyn pathology; the disruption of the autophagy-lysosome pathway (ALP) may contribute to αSyn aggregation. As a result of this, aberrant methylation takes place at 928 cytosines affecting 326 ALP genes in the appendix; in addition, widespread hypermethylation was also found in the brain of individuals with PD [60]. 

Epidemiological studies have provided evidence that exposure to organochlorine agrichemicals elevates a person’s risk for PD. A comparison of plantation workers with different terms of occupation detected seven and 123 differentially methylated loci in brain and blood cell DNA, respectively [61]. The blood of patients with dementia with Lewy bodies (DLB) shows differential methylation compared to the blood of patients with Parkinson’s disease dementia (PDD) and sets of probes show high predictive value to discriminate between variants [62]. DNA methylation patterns are established and maintained by DNA methyltransferases (DNMTs), and it was found that protein expression of DNMT1 was reduced in the cellular and mouse models of PD. Paradoxically, mRNA levels of DNMT1 were increased in these models [63].

Aberrant DNA methylation is closely associated with many aspects of the pathogenesis of PD and presents a mechanism to investigate inflammation, aging, and hematopoiesis in PD, using epigenetic mitotic aging and aging clocks. In early PD, accelerated hematopoietic cell mitosis was revealed, possibly reflecting immune pathway imbalances, which may be related to motor and cognitive progression [64]. The integration of metabolomics and epigenetics (genome-wide DNA methylation; epimetabolomics) was described after studies of the frontal lobe of people who died from PD: 48 metabolites and 4313 differentially methylated sites were identified in the primary motor cortex of people who died from PD, as compared with age- and sex-matched controls [65]. The metabolite taurine level correlated with CpG methylated sites, and bile acid biosynthesis was the major biochemical pathway to be perturbed in the frontal lobe of PD sufferers. Decreased levels of bacterially produced butyrate are related to epigenetic changes in leucocytes and neurons from PD patients and to the severity of their depressive symptoms [66].

Aberrant histone acetylation is also involved in the pathophysiology of PD. For instance, immunoblotting analyses revealed increased acetylation at several histone sites in PD, with the most prominent change observed for H3K27, a marker of active promoters and enhancers [67]. Changes in histone acetylation profile triggered by the neurotoxic mitochondrial complex II inhibitor 3-nitropropionic acid (3-NPA), were significantly different from the transcriptomic profile induced by MPP^+^ and Manganese (Mn) [68]. In a cell model of PD, αSyn significantly increased MHC-II expression, together with IFN-ɣ and IL-16 levels, which were potentiated with CUDC-907 (a dual PI3K and histone deacetylase (HDAC) inhibitor) and TMP-195 (a potent and selective inhibitor of class IIa HDAC) [69].

Histone methylation and acetylation are involved in synchronizing gene expression and protein function in neuronal cells, and manipulations of these two mechanisms influence the susceptibility of neurons to degeneration and apoptosis. Some pharmaceuticals, such as HDAC inhibitors and DNA methylation inhibitors, were developed to deal with CNS disease by targeting epigenetic components (see Section 10.10).

Noncoding RNAs consist of a very special class of epigenetic regulators. The change from viewing noncoding RNA as “junk” in the genome to seeing it as a critical epigenetic regulator in almost every human condition or disease has forced a paradigm shift in biomedical and clinical research. Small and long noncoding RNA transcripts are now routinely evaluated as putative diagnostic or therapeutic agents [70]. Long non-coding RNAs (lncRNAs) are a class of ncRNAs that have a length of 200 nt or more [71]. MicroRNA (miRNA, miR) are short non-coding RNA molecules with approximately 17–22 nucleotides in length [72]. Both lncRNA and miRNA control gene expression post-transcriptionally through either translational repression or mRNA degradation [73]. An excellent review has recently been published that highlights multiple aspects of the regulatory and diagnostic roles of miRNAs in PD: these are signaling mechanisms and epigenetic regulation; inflammation, ferroptosis, mitophagy and autophagy mediating pathways and miRNAs as biomarkers and therapeutic targets [74].

## 3. Organophosphates and Other Environmental Toxicants as a Cause of Sporadic PD

Environmental toxicants may interact with various parts of neurotransmission systems, including synthetic and degradative enzymes, presynaptic vesicles and the specialized receptors that characterize neurotransmission systems. Numerous neurotransmitters have been described in mammals, amongst them acetylcholine, amino acids, amines, peptides and gases. An important substance acting on the glutamatergic system is domoic acid, responsible for amnesic shellfish poisoning. 4-Aminobutyric acid (GABA) and glycine are inhibitory neurotransmitters and their antagonists, fipronil (an insecticide) and strychnine, respectively, are excitatory [75]. Important toxicants acting on the cholinergic system include anticholinesterases (OPs and carbamates) and substances that act on receptors, such as nicotine and neonicotinoid insecticides, including imidacloprid. Since the 1970s, organophosphates (OPs) have been common active ingredients in pesticides. OP-derived etiology of parkinsonism is rather intriguing since the etiology of PD is principally discussed in terms of a balance between cholinergic and dopaminergic neurotransmission in the striatum. Therefore, a possible relationship between exposure to OPs and alterations in the central cholinergic or dopaminergic activity was suggested by many researchers [76,77,78,79,80,81,82]. These suggestions are based on lots of cases of parkinsonism that were reported following acute, subacute and chronic exposure to OPs [76,77,78,81,83,84,85,86]. Moreover, the risk of PD was associated with rural living per se, because of the increased probability of chronic exposure to pesticides, including OPs [78]. Evidence suggests an association between chronic occupational exposure to OP pesticides and neuropsychological effects, particularly in individuals with certain paraoxonase-1 (PON-1) genotypes [87]. However, there is no consensus about the specific cognitive skills affected [88].

OPs are among the most dangerous xenobiotics with neurotoxic effects [89]. Triphasic effects and four pathological states resulting from exposure to OPs have been described: cholinergic crisis, intermediate syndrome (IMS), organophosphate-induced delayed neuropathy (OPIDP) and neuropsychic disorder caused by chronic exposure to OPs [90]. These pathologies, however, do not reflect the entire palette of comorbidity, given the many factors of a genetic, climatic, and social nature associated with a particular person. Single acute, repeated subacute, and chronic exposure to OPs can determine the development of various neurodegenerative and mental diseases, in addition to OPIDP [91]. These include dementia, attention deficit hyperactivity disorder, amyotrophic lateral sclerosis (ALS), multiple sclerosis, and also PD [92,93,94,95]. Clinical studies, supported by experiments on animal models, demonstrate the neurotoxic impact of insecticide exposure during the period of cerebral development; the developing brain is particularly vulnerable to the action of insecticides. Moreover, detoxifying systems that are highly polymorph lead to great inter-individual variability in susceptibility to neurotoxic effects [96]. The mechanism(s) by which OPs may cause degeneration of dopaminergic neurons remains elusive, though oxidative stress, neuroinflammation, axonal transport deficits, and autoimmunity have been suggested [97,98,99,100]. For example, chlorpyrifos (CPF) could inhibit cell proliferation, activate cell pyroptosis and increase susceptibility to oxidative stress-induced toxicity by elevating miR-181 through the downregulation of the SIRT1/PGC-1α/Nrf2 pathway in human neuroblastoma SH-SY5Y cells [101]. In addition, CPF exposure altered the expression of genes associated with intrinsic apoptosis, significantly elevating the expression of the pro-apoptotic mediator Bbc3/Puma [102]. Elevated autophagy-related protein expression in Bbc3-/- neuronal cultures was associated with a reduction in CPF-induced high molecular weight αSyn and tau immunoreactive protein aggregates. 

The emerging role of the gut microbiota in PD [103] and the effects of OPs on the microbiome may represent another fruitful avenue for mechanistic investigations. For example, OP diazinon, given in drinking water (4 mg/L for 13 weeks) to mice has been shown to alter the gut microbiome, the functional metagenome, and the associated metabolic profiles [104]. Interestingly, the effects were more pronounced in male than in female mice. Examples of observed effects include significant changes in bacterial genera, alterations in bile acid abundance, and a drastic decrease in taurine levels [104]. In another study, specific changes to the gut microbiome caused by diazinon involving oxidative stress pathways, fatty acids and carbohydrate metabolism, and quorum sensing systems were identified [105]. A human study by Stanaway et al. [106] examined the oral buccal microbiomes in farmworkers using pesticides and found an association between exposure to azinphosmethyl and perturbations in seven common bacterial taxa including significant reductions of *Streptococcus*.

Clinicians should raise the cognition of OP pesticide poisoning in patients with cognitive impairment, especially for patients with mild cognitive impairment, which can be easily ignored for a long time. The significance of early detection and diagnosis of PD and other neurodegenerative diseases is particularly important [107].

## 4. Mitochondrial Dysfunction in PD

The dysfunction of mitochondria and mitophagy are the earliest events in PD. Dopaminergic neurons are thought to be particularly susceptible to mitochondrial dysfunction. Besides the vital role in ATP synthesis, MCh takes part in the regulation of cellular metabolism, calcium storage and ROS balance, damage-associated molecular patterns (DAMP) production, inflammation and immunity, and programmed cell death [108,109]. Mitochondrial disorders in the brain are manifested even at the asymptomatic stage of the disease [110,111]. The first study showing that defects in mitochondrial respiration may be causal in PD came in the early 1980s: experimental inhibition of complex I (NADH-ubiquinone reductase) of the ETC by different compounds (MPP^+^, 6-hydroxydopamine, rotenone, and annonacin) was sufficient to cause the Parkinson-like symptoms [112]. In reactions occurring in the processes of oxidative phosphorylation, electrons are often lost during their transfer between complexes of the ETC and react with molecular oxygen to produce ROS. These include highly reactive free radicals, such as superoxide anion (O_2_^−^) or hydroxyl radical (OH^−^) and non-radical species, such as hydrogen peroxide (H_2_O_2_) [81]. ROS sources, such as NADPH oxidases (NOX), MCh, ER, xanthine oxidase, peroxisomes and cytochrome P450 oxidases can be responsible for cellular ROS production. In the peroxisomes, the major source of peroxisomal ROS is the process of beta-oxidation of fatty acids, with acyl-CoA oxidase (ACOX) being the principal enzyme. Numerous other enzymes also contribute to the production of peroxisomal ROS: xanthine oxidase (XO), D-amino-acid oxidase (DAO), D-aspartate oxidase (DDO), L-pipecolic acid oxidase (PIPOX), L-α-hydroxyacid oxidase (HAO), and polyamine oxidase (PAOX) [113]. 

MCh serves as both a source and a target of ROS. ROS accumulation can cause oxidative damage to cellular components. MCh are vulnerable targets for oxidative stress. The accumulation of ROS can result in the release of cytochrome *C*, inducing caspase-mediated apoptosis, a process of programmed cell death crucial for cell and tissue homeostasis [114]. The initial ROS is mainly O_2_^−^, generated by complexes I and III, which can be rapidly converted into H_2_O_2_ by the superoxide dismutase (SOD) [115]. H_2_O_2_ is a stable and widespread form of cellular ROS, and its role as a second messenger is extremely important in cellular redox communications. H_2_O_2_ has a longer cellular half-life (~1 ms) and a concentration of ~10^−7^ M under cellular homeostatic conditions [113]. It functions as an important signaling molecule involved in many cellular processes. H_2_O_2_ modulates the activity of target proteins through the reversible oxidation of critical protein thiols, thus altering the activity of enzymes, kinases, phosphatases and transcription factors in the MCh, cytosol, or nucleus. ROS-mediated oxidation can lead to the formation of disulfide bonds and the stabilization of the protein structure [116]. The intensity of oxidative stress depends on the balance between ROS production and antioxidant defense systems and is associated with the pathogenesis of PD [117,118]. The oxidatively modified αSyn is prone to aggregation [11]. The main antioxidant proteins are SOD enzymes, which include cytoplasmic Cu-Zn-SOD (SOD1) and mitochondrial Mn-SOD (SOD2). In addition, glutathione peroxidase (GPx), glutathione-S-transferase Pi (GST-Pi), metallothionein-3 (MT3), ferritin heavy chain (FHC), and dihydrodiol dehydrogenase (DDH1 or AKR1C1) also play an important role in antioxidant processes [119].

Along with antioxidant enzymes, MCh rely on mitophagy, which is a specialized type of autophagy that mediates the selective removal of damaged MCh from cells, with the net effect of dampening the toxicity arising from these dysfunctional organelles [8]. There are three pathways at the crossroads of mitophagy. The first, ubiquitin-mediated mitophagy, involves the recruitment of PINK1 and Parkin to the outer mitochondrial membrane (OMM), which promotes the sequestration of damaged MCh into phagophores called mitophagosomes; the latter subsequently fuse with lysosomes, where the cargo is degraded. It is a multi-step process that ensues following the loss of mitochondrial membrane potential. The second, receptor-mediated mitophagy involves the direct binding of mitophagy receptors NIX/BNIP3L or FUNCD1 to LC3 on the autophagosomes, which then deliver the engulfed damaged mitochondria to the lysosome. In the third pathway, which is known as lipid-mediated mitophagy, cardiolipin is externalized from the inner mitochondrial membrane (IMM) to the OMM, where it binds to LC3 on mitophagosomes [8]. Impairment of mitophagy was observed in several PD models and found in the brains of PD patients [120,121].

## 5. α-Synuclein in Health and Parkinson’s Disease

**Figure 2:**
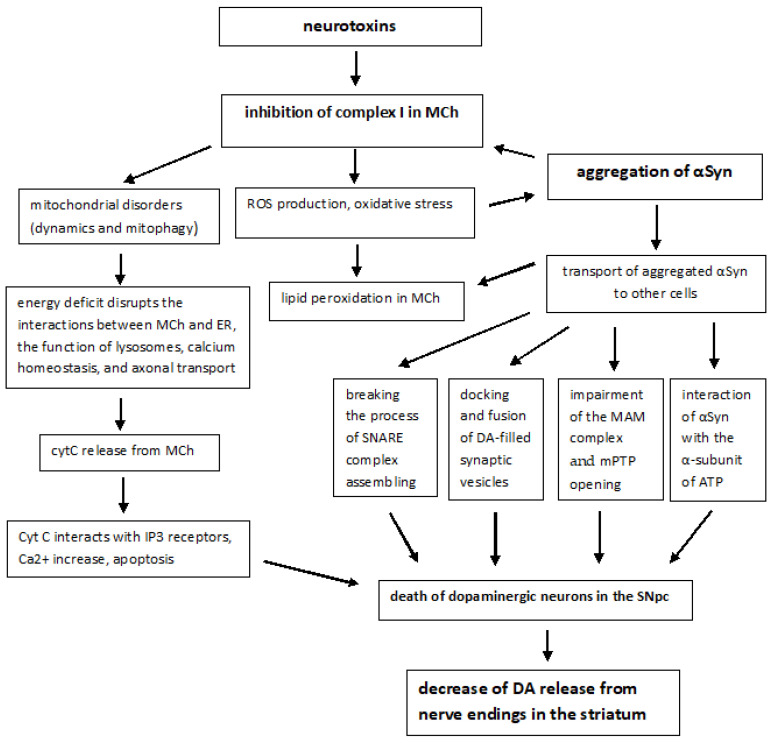
Molecular interactions and cause-effect pathways in PD. Key links or events are in bold. All the details are described in Section 5.

PD is characterized by abnormal accumulations and aggregation of αSyn within neuronal cells. αSyn accounts for 1% of the total protein content in neurons and is a small protein with a molecular weight of 14 kDa, consisting of 140 amino acids [122,123]. Normally, it directly interacts with many proteins involved in DA homeostasis. On the other hand, aggregated αSyn impairs many processes and mediates neurodegeneration. The *N*-terminal region is amphipathic and is responsible for the interaction of αSyn with lipid membranes [124]. The central domain, known as the non-amyloid-β component (NAC) contains a highly hydrophobic motif essential for αSyn aggregation [91,125]. The *C*-terminal region is highly negatively charged [126], due to negatively charged amino acids alternating by proline residues. In its native state, αSyn is present as a tetramer with an ordered structure formed of alpha-helices [127,128]. αSyn directly interacts with membrane lipids, synaptic vesicles, proteins of the SNARE complex, proteins involved in DA homeostasis, proteins involved in calcium regulation, and the catalytic subunit of phosphatase 2A (PP2A) (Figure 2) [129,130].

### 5.1. Role of αSyn in DA Synaptic Transmission in the Normal State

αSyn is highly expressed in both the central and peripheral nervous system and it is particularly enriched in the nerve terminals [131]. In the presynaptic terminals of the central nervous system, αSyn is known to interact with many partners, such as monoamine transporters, cytoskeletal components, lipid membranes, chaperones, and proteins associated with synaptic vesicles. αSyn interacts predominantly with membranes of high curvature, abundant in acidic phospholipids. The C-terminal domain of αSyn mediates the interaction with the soluble NSF protein of the SNARE complex. In the presence of acidic membrane phospholipids or highly curved membranes the N-terminal region of αSyn folds into an alpha helix at interaction with membranes and plays a role in the docking, fusion, and clustering of synaptic vesicles, in particular those filled with DA [132,133]. The binding of intracellular Ca^2+^ to the C-terminus of αSyn affects the binding of αSyn to the lipid membrane, thereby modulating synaptic vesicle interactions. Thus, the αSyn protein participates in the assembly of SNARE complexes as a chaperone and regulates membrane fusion. Under conditions of excess Ca^2+^, αSyn undergoes aggregation. Pathological αSyn molecules are neurotoxic structures that mediate neurodegeneration and propagate between neurons (Figure 2) [134,135]. 

Through reuptake in the presynapse, DA can be recruited for exocytosis. Under conditions of ATP deficiency, the neurotransmitter recycling process is disrupted. With the formation of fibrils of αSyn, the packaging of DA into vesicles deteriorates and DA accumulates in the cytosol. Excess DA can be metabolized by MAO to the toxic metabolite DOPAL, which promotes oxidative stress, ROS formation, mPTP opening, and death of dopaminergic neurons [136].

### 5.2. Prion-like Effect of αSyn

Under pathological conditions, soluble αSyn forms β-sheet oligomers (protofibrils), which are converted into amyloid fibrils and eventually deposited in Lewy bodies. It was previously reported that pathological conditions and aggregation of αSyn are caused by different factors, such as pesticides, herbicides, heavy metals, polycations, histones, organic solvents, heat shock proteins (Hsp), oxidative stress, post-translational modifications, etc. [137,138]. Studies of the accumulation of LB in the post-mortem brain of patients with PD from the early to late stages of the disease have shown that LB is first found in the olfactory bulb and ENS [139]. 

αSyn fibrils can spread from cell to cell, contributing to pathogenicity [140,141]. LRP1 was recently shown to be a key regulator of αSyn neuronal uptake, as well as an important mediator of αSyn spread in the brain [17]; previously it was mainly known to regulate the spread of tau proteins [142]. At the same time, the expression of LRP1 is lowered in the brain endothelial cells of PD patients, breaking the maintenance of the BBB [143]. In addition to the release, internalization and receptor-activating mechanisms, tunneling nanotubes directly connect two adjacent cells and participate in the cell-to-cell transfer of pathological αSyn assemblies [144].

### 5.3. αSyn Inhibits Formation of the TOM Complex

More than a thousand human mitochondrial proteins are encoded by the nucleus and synthesized on cytoplasmic ribosomes [145], and therefore, must be imported into MCh via highly conserved protein translocation pathways. The identification and uptake of these proteins are mediated on the mitochondrial surface by components of the TOM (the translocase of the outer membrane) complex. The TOM core complex (TOM-CC) consists of five components: TOM40, TOM22, TOM7, TOM6, and TOM5 [146,147]. It was shown that oligomeric, and post-translationally modified αSyn (oxidized, or DA modified), but not monomeric or nitrated αSyn, binds to a subunit TOM20 and prevents its association with TOM22, a key step in the formation of the TOM complex that is necessary for protein import [148]. TOM20 overexpression has been shown to reverse αSyn-induced dopaminergic neurodegeneration over a 12-week time period [149,150]. Later studies in vivo have shown that TOM20 overexpression restores levels of nuclear-encoded mitochondrial proteins, even in the face of continued αSyn overexpression, and protects these neurons against degeneration [151]. Therefore, the blockade of approximately 99% of mitochondrial protein import by toxic species of αSyn may be an early and important contributing factor to dopaminergic neurodegeneration [152,153].

### 5.4. αSyn Participates in Calcium Transfer between ER and Mitochondria

The MAM complex is the interface between MCh and the ER that performs several important functions, including Ca^2+^ signaling, cholesterol and phospholipid metabolism, apoptosis, ER stress, and mitochondrial division [154]. 

The MAM complex provides direct Ca^2+^ transport from the ER to the MCh, creating a high local calcium concentration necessary for the operation of the MCU, which has a low affinity for calcium. Normally, the accumulation of Ca^2+^ in the mitochondrial matrix stimulates respiration and ATP production through the activation of mitochondrial enzymes, such as pyruvate dehydrogenase (PDH), isocitrate dehydrogenase (ICDH), and oxoglutarate dehydrogenase (OGDH) [155]. Additionally, mitochondrial Ca^2+^ regulates the activity of complexes III and V of the electron transport chain [156,157]. However, excessive accumulation of calcium ions inhibits these functions and stimulates the production of ROS. Moreover, large Ca^2+^ spikes in MCh, especially under conditions of oxidative stress and membrane depolarization with high Pi and depletion of adenine nucleotides, can cause the opening of a multicomponent megachannel, mPTP, with variable conductivity (up to 1.5 nS) and induce MCh-dependent apoptosis. Upon the induction of apoptosis, cytochrome *c* released by MCh binds to InsP3R (inositol 1,4,5-trisphosphate receptors) and blocks Ca^2+^-dependent inhibition of InsP3R [158], which leads to an increase in the flow of Ca^2+^ ions from the ER and additional release of cytochrome *c* (Figure 2).

Wild-type αSyn localizes to MAMs, contributes to regulating ER–mitochondria communication and influences the transfer of Ca^2+^ between ER and MCh. Cell culture studies showed that wild-type αSyn overexpression increased MAM and increased mitochondrial uptake of Ca^2+^ from the ER, promoting SN neuronal death and PD progression [159,160]. 

### 5.5. αSyn and Mitochondrial Voltage-Dependent Anion Channel (VDAC) 

VDAC is a key player in many mitochondrial processes such as signaling, apoptosis, and calcium homeostasis. VDACs are responsible for ~90% of the outer mitochondrial membrane’s overall permeability [161]. VDAC’s main function is to maintain the metabolic cross-talk between the mitochondria and the rest of the cells, allowing the diffusion of essential hydrophilic metabolites up to 5 kDa in size [162]. VDAC represents the main route for newly synthesized mitochondrial ATP, and the entry route to the organelle for cytosolic ADP, pyruvate, glutamate, succinate, and Krebs’s cycle intermediates. Ions, including Cl^−^, Na^+^, K^+^ and Ca^2+^, as well as NAD^+^/NADH also use VDAC as a mitochondrial gateway [163]. VDAC is not only a channel for these connections but also regulates their fluxes [164,165]. Using VDAC reconstitution experiments, it was shown that VDAC regulates the fluxes of metabolites and calcium across the outer mitochondrial membrane by briefly switching between states in which its permeability to metabolites and calcium changes, i.e., VDAC’s open state facilitates flux of anionic metabolites but maintains a low calcium flux and vice versa [165,166]. 

High-affinity interaction of αSyn with the VDAC was established, at which nanomolar concentrations of αSyn reversibly blocked the VDAC, and αSyn also moved through this channel [167]. The mechanism of αSyn docking to VDAC appears to be recruitment to the membrane containing VDAC, followed by voltage-dependent entry of the C-terminal domain into the VDAC pore [168]. αSyn not only crosses the mitochondrial outer membrane but also targets respiratory complexes leading to defects in bioenergetics [169]. Overexpression of αSyn can cause the degeneration of dopaminergic neurons through its interaction with mitochondrial VDAC1, subsequent mPTP activation, mitochondrial uncoupling, and cell death [170].

By changing the permeability of VDAC1 to Ca^2+^, αSyn modulates the pathway by which calcium ions are transported from the ER to the MCh [171]. In fact, as a result of αSyn binding, the selectivity of VDAC1 for calcium increases, which enhances the flow of Ca^2+^ through the channel [172] (Figure 1 and Figure 2). Thus, the important role of VDAC in energy metabolism and calcium homeostasis has made it a potential therapeutic target in diseases such as PD [173,174,175].

### 5.6. αSyn and Control of Mitochondrial Bioenergetics

The control of mitochondrial bioenergetics is another important function that αSyn performs normally. In mice lacking αSyn, a decrease in the activity of ETC complexes I and/or III was observed, which was associated with disorders in the lipid composition of MCh [176]. Moreover, MCh obtained from α, β, and γ synuclein knockout mice were shown to be characterized by uncoupled mitochondrial respiration [177]. The addition of monomeric αSyn to isolated brain MCh increased the efficiency of ATP synthesis due to the direct interaction between αSyn and the α-subunit of ATP synthase [177]. On the other hand, αSyn oligomers not only disrupt complex I-dependent respiration but also induce selective oxidation of the beta subunit of ATP synthase and lipid peroxidation in MCh (Figure 2). Oxidation increases the probability of mPTP opening, causing mitochondrial swelling and ultimately cell death. It should be noted that the inhibition of oligomer-induced oxidation prevents pathological mPTP induction [175]. These data point to the important role of monomeric αSyn as a mitochondrial bioenergetic regulator.

## 6. Physiological Features of Neurons and Vulnerability of Nervous Systems

### 6.1. Nigrostriatal Neurons

The high-energy requirements of the brain support synaptic neurotransmission, action potential firing, synapse development, maintenance of brain cells, neuronal plasticity, and cellular activities required for learning and memory [178,179]. Dopaminergic neurons consume  ~20 times more energy compared to other neurons due to their anatomical structure: long and branched axons, more neurotransmitter release sites and their pacemaker activity [180,181]. Additionally, dopaminergic neurons are characterized by a high basal rate of mitochondrial oxidative phosphorylation, an elevated level of basal ROS production and increased mitochondrial oxidative stress [182]. In the absence of external stimulation of the SN, DAergic neurons generate broad (~2–3 ms) action potentials (AP) at a relatively slow rate (2–10 Hz) [150,183]. The rhythmic pacemaker activity is due to the properties of the pore-forming subunit of Cav1.3 of the L-type Ca^2+^-channels that regulate the basal level of DA in the striatum [184,185]. DAergic neurons have large cytosolic oscillations of Ca^2+^ concentration ([Ca^2+^]), which play a key role in helping the neurons meet their bioenergetic needs, but they are also linked to cellular stress and vulnerability with aging and PD [186]. The well-known fact is that cytosolic [Ca^2+^] oscillations in DAergic neurons of the SNpc initiate Ca^2+^-entry into MCh and stimulate Ca^2+^-dependent dehydrogenases [155,185]. Subsequent activation of respiration in the absence of a high rate of ATP production leads to mitochondrial hyperpolarization and increased ROS production [182,186]. The rhythmic pacemaker activity of dopaminergic neurons was suggested to be one of the reasons for the high energy demand and vulnerability of these cells. It was shown that a higher basal rate of mitochondrial oxidative phosphorylation and an elevated level of basal ROS production characterized nigral DAergic neurons compared to DAergic neurons of the VTA (ventral tegmental area) [182]. It should be noted, that factors contributing to the vulnerability described in the DA neurons of SNpc, i.e., slow pacemaking, cytosolic Ca^2+^ oscillations, low intracellular Ca^2+^ buffering [187], and elevated levels of mitochondrial oxidant stress, were also found in other vulnerable non-DA neurons [188]. 

### 6.2. Enteric Nervous System and Enteroendocrine Cells

αSyn pathology in PD is not limited to the brain and is also observed in the peripheral nervous system (PNS) including the ENS [189]. Some postmortem findings indicate that the olfactory bulb and the ENS are among the first areas affected by Lewy pathology, and those regions that are anatomically interconnected become gradually involved in the pathogenesis (Braak’s hypothesis). Many intestinal neurons, especially those involved in peristalsis, are also autonomous and probably permanently active, making them just as vulnerable. All enteric neurons have unmyelinated axons and many of them have multiple synaptic endings, features that may also increase their vulnerability to PD and may facilitate the release and reuptake of αSyn and its spread through associated ganglia [190]. Experimental evidence has previously been presented suggesting that in PD, αSyn accumulates in enteric neurons prior to motor symptoms and various forms of αSyn can spread from the gut to the brain; aggregated αSyn is transported via microtubules [47]. There is growing evidence indicating that abnormal αSyn can spread to neighboring brain regions and cause the aggregation of endogenous αSyn in these regions in a “prion-like” manner [191]. It should be noted that prions can cause CNS disease through multiple transmission routes, including intracerebral, intraperitoneal, intravenous, and oral routes [192].

Thus, the early symptoms of PD manifest as impaired function in tissues such as the GI tract, olfactory system, and brainstem (a dual-hit hypothesis) [44,193]. In addition, it has been suggested that abnormal αSyn first develops in neuronal enteroendocrine cells (EECs) and then enters the CNS [194,195]. EECs possess many neuron-like properties including αSyn expression. By facing the gut lumen and being directly connected with αSyn-containing enteric neurons in a synaptic manner, EECs form a neural circuit between the GI tract and the ENS. In both the EECs line STC-1 and the neuronal cell line SH-SY5Y, αSyn pre-formed fibrils (PFFs) induced intracellular Ca^2+^ oscillations in an extracellular, Ca^2+^ source-dependent manner and triggered αSyn fibrils internalization by endocytosis [196]. The spread of αSyn PFFs from enteroendocrine to neuronal cells is dependent on physical cell-to-cell contact and on Rab35 GTPase; the inhibition of Rab35 increases the clearance of αSyn fibrils by redirecting them to the lysosomal compartment. 

## 7. Gastrointestinal (GI) Tract in Pathogenesis of PD

### 7.1. Gut Microbiota May Influence αSyn Aggregation

Various metabolites produced by gastrointestinal microorganisms can influence aSyn aggregation and cause neurodegenerative processes [197,198]. There are around 100 trillion bacteria that reside in the GI tract and in essentially every part of the human body, from the nasal cavities to the urogenital system. These microbes whose genome is believed to be nearly 100-fold the size of the human genome, live in symbiosis with the smaller population of eukaryotic cells in the body and play important roles in its development and homeostasis [199,200]. An imbalance in the intestinal microbiota leads to the appearance of damaging factors in MCh, which causes neuropsychiatric diseases and neurodegenerative processes [161,162]. A complex system of cross-interactions between the gut microbiota, the ENS and the CNS has been proposed to describe the phenomenon [201]. The gut microbiota converts substrates into various metabolites including short-chain fatty acids (SCFA), niacinamide (NAM), bile acids (BA), and neurotransmitters [202,203]. Studies have shown that SCFA and extracellular fibers, such as curli produced by gastrointestinal microorganisms, can influence αSyn aggregation and motor dysfunction [204]. The analysis of human pathology led Braak to postulate that αSyn pathology could spread from the gut to the brain, via the vagus nerve [139,180], and PD can also be caused by a pathogen that enters the body through the nasal cavity and is then swallowed and reaches the intestines, causing Lewy pathology in the nose and digestive tract [45]. It has also been shown that the intragastric administration of rotenone to mice induces oxidative stress and the accumulation of phosphorylated αSyn in the ENS and dorsal motor nucleus of the vagus (DMV), which ultimately leads to neurodegeneration in the SNpc [205,206]. Introduction of αSyn fibrils into the gut converts endogenous αSyn into pathological species that spread via the vagus nerve to the brain and cause PD-like motor and non-motor symptoms. Vagotomy and αSyn deficiency prevented αSyn-induced neuropathology and neurobehavioral disorders [207].

### 7.2. αSyn Accumulates in the GI Tract

Neurotransmitters play a significant role in GI physiology. DA, γ-aminobutyric acid (GABA), and serotonin (5-hydroxytryptamine, 5-HT) influence gut motility, nutrient absorption, the innate immune system in the GI tract, and the microbiome [208]. Neurotransmitter levels may be altered by GI disturbances in patients with PD. Constipation, a common symptom of PD, may appear several years earlier than motor symptoms. It has been shown that αSyn first accumulates in the submucosal nerve plexus of the intestine. The ENS and the vagus nerve can be affected already in the prodromal phase of the disease [209]. The intestinal barrier includes a layer of epithelial cells connected by transmembrane proteins that are associated with various tight junction proteins of the plasma membrane, such as ZO-1 (zonula occludens-1), whose function is to anchor the actin microfilaments of the cytoskeleton inside the membrane [210]. Under physiological conditions, the integrity of the intestinal barrier prevents bacteria and LPS from contacting epithelial cells. A decrease in ZO-1 expression disrupts the permeability of the barrier. The disrupted intestinal barrier allows bacteria and LPS to penetrate the epithelium into the bloodstream under the intestinal mucosa. The expression of ZO-1 in patients with PD is significantly lower than in healthy people; the level of LPS in the colon and plasma of patients with PD is significantly increased, which leads to the activation of a number of inflammatory reactions [210].

## 8. Endothelial Cells (ECs) and Blood Vessel Damage in Pathogenesis of PD

Vascular damage, along with misfolded proteins and inflammatory responses, is an indispensable pathological feature of neurodegenerative diseases. αSyn fibrils can invade the CNS after a certain period of time following their oral or intravenous injection, which is followed by the development of neuropathology and disease [211]. It is believed that vascular factors accelerate the onset of motor and cognitive impairment in the early stages of PD [212]. Studies using non-invasive MRI in a heterogeneous population of patients with PD revealed a decrease in cerebral blood flow by 20–40% in various areas of the brain [213,214]. There is evidence for the formation of filamentous vessels in the capillaries of the brain in PD: in fact, these are collapsed basement membranes of capillaries that lack endothelium and have lost the function of blood passage [215]. In addition to the damage to the ECs, thickening of the basement membrane, vacuolization and degradation of pericytes were found in the cerebrocortical microvessels of patients with PD [216].

There are no vascular smooth muscle cells in the capillaries of the brain, but they are partially covered with pericytes that have contractile functions [217,218]. In addition, brain ECs regulate vascular tone by releasing vasodilators including nitric oxide (NO), epoxyeicosatrienoic acids (EET), PGE2 and prostacyclin, as well as vasoconstrictors such as endothelin-1, thromboxane A2 and prostaglandin F2α [219,220]. Vascular defects may occur at an early age, affecting brain maturation, but may also appear at a later age, participating in neurodegenerative processes, including PD (Table 1). More and more recent data indicate that vascular disorders largely determine the pathogenesis of neurological diseases throughout life [221,222,223]. 

### 8.1. αSyn, ECs and Vascular Factors in PD

Expression of αSyn in ECs of vessels supplying the brain and peripheral organs was noted as early as the mid-1960s [251]. No direct effect of αSyn on EC has been shown. However, the thickening of the basement membrane was found in the capillary beds in the brains of patients with PD [215,216,252]. Experimental and clinical studies revealed a reduced level of tight junction (TJ) proteins [253,254]. Such changes reduce the efficiency of molecular exchange between the brain parenchyma and blood vessels, which increases the vulnerability of neurons to oxidative stress and promotes the accumulation of modified proteins and low molecular weight metabolites. The TJ of the brain ECs provide barrier functions and determines the special purpose of EC in maintaining homeostasis and in the development of neurodegenerative diseases [219,255]. Along with a decrease in the expression of TJ in the brain of patients with PD, suppression of angiogenesis was found, which indicates endothelial dysfunction. These changes were noted mainly in the SNpc, locus coeruleus (LC) and caudate nucleus (NC, nucleus caudatus), and to a lesser extent in the cerebral cortex [252,256,257]. 

Exosomal αSyn induces endogenous soluble protein oligomerization in recipient cells. In addition, erythrocyte-derived exosomes containing αSyn induce microglial activation in PD patients [258]. The concentration of αSyn in erythrocytes is about 1000 times higher than in cerebrospinal fluid. Erythrocyte vesicles (EVs) that cross the BBB localize adjacent to brain parenchyma microglia and induce or enhance microglial inflammatory responses even though they do not contain αSyn. However, if EVs are derived from the erythrocytes of PD patients, then they elicit a stronger response compared to EVs from healthy subjects [258]. PFFs suppress the expression of occludin and ZO-1, which leads to an increase in paracellular transport between ECs, but endothelial dysfunction and the release of pro-inflammatory cytokines from ECs are not observed [254]. Inflammation caused by pathological αSyn isoforms causes EC activation, which manifests itself in EC contraction, increased paracellular permeability, and the generation of pro-inflammatory cytokines and chemokines. First, these factors cause an increase in local blood flow, then BBB dysfunction occurs; the extravasation of protein-rich exudate and the recruitment and activation of circulating leukocytes further contributes to neuroinflammation [259,260]. The impact of only oligomeric αSyn, but not monomeric or fibrillar, leads to a significant disruption of the integrity of the BBB. This process involves the expression and release of VEGFA and NO from astrocytes exposed to oligomeric αSyn [261]. Chronic effects of TNF-α and IL-1β on ECs associated with αSyn deposition and observed in PD patients and in animal models [262] cause NF-κB and AP-1 activation, and increased expression of VCAM-1 and ICAM-1, creating the basis for further neuroinflammation and neurodegeneration [260].

At the same time, αSyn inhibits the agonist-induced release of von Willebrand factor (vWF) and translocation of P-selectin from Weibel–Palade bodies (WPB) into ECs [263]. Agonists, such as thrombin, VEGF, histamine and ROS cause an increase in intracellular calcium, which binds calmodulin and triggers the translocation of Ral-specific guanine exchange factor (RalGDS) from the cytosol to the plasma membrane, activating membrane-bound RalA (small GTPase and substrate for RalGDS) by exchanging GDP for GTP [264,265]. After that, RalA-GTP interacts with the exocyst and a multiprotein complex is formed that facilitates the transport of vesicles to the plasma membrane and exocytosis [266]. When ECs are exposed to forskolin or norepinephrine, the cAMP level increases and protein kinase A (PKA) is activated, which also induces RalGDS translocation to the membrane [263,265]. Upon activation of these pathways, αSyn binds to both RalGDS and β-arrestin, thereby enhancing their interaction, inhibiting their dissociation and translocation to the plasma membrane, and preventing WPB exocytosis [260]. Considering that WPB also contain other important mediators of inflammation and hemostasis (interleukin-8, eotaxin-3, endothelin-1, angiopoietin-2, etc.) [265], αSyn has a significant effect on vascular homeostasis during the inflammatory response and thrombosis [260]. In this regard, the question of the primacy of pathological changes in PD turned out to be not so simple and unambiguous. 

Lymphocytes are also involved in the pathogenesis of PD, mainly due to the infiltration of T cells with increased expression of LFA1, a receptor for ICAM-1 on ECs [267]. T-cell reactivity specific to αSyn is more pronounced at the early stage of PD [268]. In this regard, it should be noted that the primary cause of an increase in the level of αSyn may be stroke, followed by the interaction of Treg cells with ICAM-1 on microvessels and platelets, which causes vascular dysfunction [269,270]. When the cytokine microenvironment in vivo undergoes inflammatory changes, Th17 cells can transform into a highly pro-inflammatory pathogenic phenotype, break through the BBB and recruit more inflammatory cells to participate in neuroinflammation [271].

### 8.2. Neurovascular Unit, Angiogenesis and the BBB Dysfunction in PD

Although neurodevelopmental disorders (autism, schizophrenia, Down’s syndrome) and neurodegenerative diseases (multiple sclerosis, Huntington’s, Parkinson’s, and Alzheimer’s) have different clinical features at different stages of life, they are characterized by similar vascular anomalies [214]. One of the key pathogenetic features of age-related neurodegenerative diseases (primarily AD, PD, and ALS) is a violation of the integrity of the BBB [257]. The anatomical substrate for the regulation of cerebral blood flow and the BBB is a multicellular system composed of neurons, astrocytes, microglia, pericytes, and ECs, known as the neurovascular unit (NVU) [272]. 

Cerebral circulation disorders in PD are associated with impaired BBB permeability [273]. Loss of BBB integrity is observed on an increasing scale in the aging brain. Among the evidence of BBB disruption in PD is a significant increase in albumin and IgG in the cerebrospinal fluid [274], and the extravasation of erythrocytes, hemoglobin and fibrin in the striatum of patients with PD [257]. Human studies are not as numerous, but are quite convincing in that BBB disruption is increased in PD compared with patients with ischemic disorders without clinical signs of PD. In patients with PD, dysfunction of the BBB with increased permeability in the post commissural putamen, SN and posterior cortical regions was revealed [273,275,276]. In animal studies, there is also a lot of evidence of a violation of the integrity of the BBB in SN [277,278,279,280,281]. Dysfunction and increased permeability of the BBB may be due to the accumulation of αSyn in ECs [237]. αSyn crosses the BBB in two directions [282], and the transport of αSyn through the BBB into the brain parenchyma increases after LPS-induced BBB disruption, which may contribute to the development of PD pathology [283]. The BBB of the striatum is especially vulnerable to ischemic, osmotic, or other stressors. According to available data, the loss of BBB integrity occurs in the striatum before the onset of pathology; from this, it follows that the presynaptic endings of the axons of striatal afferents are exposed to blood-borne substances [257,284,285]. 

Other data also support the endothelial component of the pathogenesis of PD, according to which BBB dysfunction, accompanied by pathological activation of pericytes, precedes the onset of neuronal degeneration in a mouse model of PD [237]. In this case, VEGF plays an important role: on the one hand, it performs a neuroprotective function in PD by acting on dopaminergic neurons [245]; on the other hand, an excess of VEGF leads to an increase in vascular permeability, a violation of the integrity of the BBB, which can become the main pathogenetic factor in various neurodegenerative diseases, including PD [286]. In the SN of patients with PD, the level of VEGF is increased, which causes angiogenesis and enhances the permeability of the BBB [287]. In this regard, it is interesting to note that VEGF arose during evolution as a signal that initially affects nerve cells, but later became known as a blood vessel growth factor [286,288].

Angiogenesis is known to be an adaptive response to cerebral hypoxia or ischemia and is regulated by basement membrane proteins and integrin receptors [289]. The expression of the αvβ integrin receptor is increased not only in angiogenic vessels but also in cerebral vessels in patients with PD and sporadic dementia with Lewy bodies (incidental LB disease, iLBD); this suggests that immature vessels formed in the brains of PD patients contribute to neuroinflammation by facilitating the infiltration of peripheral immune cells, inflammatory or toxic factors [256]. In an animal model of PD, co-localization of areas of penetration into the brain parenchyma of an intravascular indicator with new vessels expressing integrin β3 indicates the presence of both angiogenesis and BBB impairment [290]. According to one of the classifications of the gradual development of PD [139], patients with iLBD are at an early stage of the disease, when LB are detected only in the LC and SN [256]. Thus, the presence of angiogenesis in patients at the iLBD stage suggests that vascular dysfunction may precede and/or contribute to the progression of neuroinflammation and neurodegeneration, and not vice versa. This is further supported by evidence that αSyn-coupled angiogenesis and downregulation of TJ protein expression are often observed regardless of inflammation [254,256]. A characteristic sequence of processes in neuroinflammation is the activation of glial cells (microglia and astrocytes), their release of proinflammatory cytokines, neurotoxicity, and neuronal dysfunction. Recently, however, more and more evidence has emerged that indicates the importance of BBB disruption in the initiation and development of neuroinflammatory processes leading to neurodegeneration. Violation of the integrity of the BBB leads to the penetration of immune cells or plasma proteins into the brain parenchyma, followed by the launch of inflammatory processes in the brain. The integrity of the BBB is influenced by many factors, both from the side of the brain parenchyma and from the blood plasma, the role and interaction of these factors is currently one of the principal problems of molecular neurology [291,292]. Thus, pathological αSyn isoforms cause increased BBB permeability and precede neuroinflammation and neurodegeneration.

## 9. Phagocytic Cells and Glymphatic Transport in PD 

The integrity of the BBB and inflammation are regulated by microglial cells. Microglia plays an important role in the response to infection and brain damage, as well as in the development of various neurological diseases [293]. These resident brain macrophages help regulate brain function by removing dying neurons, and non-functioning synapses, and producing ligands that support the survival of neurons [294]. Microglia protect the brain from excessive activation in normal and diseased conditions by responding to an excess extracellular level of ATP and acting similarly to inhibitory neurons [295]. Perivascular microglia play a dual role in the pathogenesis of PD: with a slight violation of the BBB, microglial cells maintain the integrity of the BBB due to the synthesis of claudin-5, a TJ protein; but with a prolonged process of inflammation, the same microglial cells increase the permeability of the BBB due to phagocytosis of the legs of astrocytes [296]. In addition, microglia exert a biphasic effect on the proliferation of brain ECs by changing the balance of TNF-α and TGF-β: the anti-inflammatory cytokine TGF-β maintains ECs at rest, while the pro-inflammatory TNF-α induces ECs proliferation [297].

CNS border-associated macrophages (BAM) are a special population of cells that control the “wrong” proteins (βA, αSyn) and, if necessary, get rid of them. In pathological conditions such as cerebral amyloid angiopathy (CAA), AD and PD, BAM may be involved in the clearance of toxic βA and αSyn [298]. Removal of perivascular macrophages (PVM) leads to increased expression of VCAM-1, T-cell infiltration, and increased levels of αSyn [299]. Pericytes and astrocytes are involved in αSyn transfer between the NVU cells, suggesting a possible role for non-neuronal cells in the spread of αSyn pathology in PD [300,301]. Recent studies have shown that αSyn activates pericytes, stimulating their release of pro-inflammatory mediators and enhancing BBB dysfunction [226,237].

αSyn is cleared from the brain via extracellular drainage pathways that include glymphatic transport and the meningeal lymphatic system [302]. Drainage disturbance contributes to the accumulation of αSyn and its aggregation in SNpc. In addition, recent studies have shown that aquaporin 4 (AQP4), the dominant water channel protein in the brain, is involved in the development of PD. Decreased expression of AQP4 in AQP4+/- mice accelerates the pathological accumulation of αSyn, and the loss of dopamine neurons, and contributes to behavioral disorders. With a decrease in AQP4 expression, the drainage of macromolecules from the brain parenchyma through the glymphatic pathway slows down [303].

An increase in the perivascular space of the basal ganglia (BG-PVS) is an indicator of the progression of motor disorders in PD [304]. Diffusion tensor image analysis along the perivascular space (DTI-ALPS) of patients with PD revealed enlarged spaces of the glymphatic system in them, which indicates weakened lymphatic drainage in the CNS and accumulation of αSyn in the cerebrospinal fluid [305,306]. Recent data from the Taoka group indicate that PD has the characteristics of interstitial fluidopathy of the CNS, and the expansion of the perivascular space of the CNS may occur as a response to the accumulation of αSyn in it [307].

## 10. Treatment and Optimization of the Condition of Patients with PD

### 10.1. Traditional Therapy of PD

The current clinical approach to PD focuses on symptomatic management. Therapy that can stop or slow the progression of neurodegeneration has not been developed, and this is largely due to a lack of understanding of the molecular mechanisms underlying the disease. Options for ablative surgery include pallidotomy (destruction of the globus pallidus), thalamotomy (destruction of the thalamus), and subthalamotomy (destruction of the subthalamic nucleus). Operations can be uni- or bilateral and are aimed at reducing excessive inhibition from the globus pallidus and SN [36,308]. The possibilities of drug therapy for PD are limited and are mainly aimed at increasing the level of DA through the introduction of the physiological precursor of dopamine 1-3,4-dihydroxyphenylalanine (L-DOPA or levodopa), which crosses the BBB and is metabolized into DA. This therapy was proposed in 1961 [309], but it is still the gold standard for the treatment of PD, which is used as a substitute for DA loss in the striatum [310]. L-DOPA is only effective in the early stages of the disease and provides only symptomatic relief with many side effects [111]. Despite its effectiveness in the treatment of motor symptoms, L-DOPA therapy causes the development of motor complications known as dyskinesia [311,312]. Moreover, L-DOPA does not counteract non-motor symptoms, which sometimes lead to the same disability as motor ones [313]. Moreover, high concentrations of L-DOPA as well as DA may induce excessive ROS production and oxidative stress, which can be reduced by dietary supplements like flavonoids and carotenoids [314,315]. 

L-DOPA loses its effectiveness over time. PD is a disease that affects multiple mediator pathways in the brain, so even when low DA problems are solved with L-DOPA, problems caused by low acetylcholine levels are not solved [36]. Among the first pharmaceuticals for the treatment of PD were anticholinergic compounds that restore the balance between DA and acetylcholine levels disturbed in PD [36,316]. Although these drugs have been largely replaced by L-DOPA and other centrally acting dopaminergic agonists, they have remained in the arsenal for the treatment of PD. This class includes benztropine, biperiden, diphenhydramine, donepezil, ethopropazine, orphenadrine, procyclidine, and trihexyphenidyl [36,317]. They are typically used in tremor-dominated PD and usually in combination with L-DOPA [318].

DA-like agonists bind to dopaminergic postsynaptic receptors and trigger the same signal as DA. This group includes pergolide, pramipexole dihydrochloride, ropinirole hydrochloride, rotigotine and apomorphine hydrochloride [319]. Apomorphine is a non-selective D1 and D2 receptor agonist and is used as a drug in patients with PD; it is considered the only drug comparable to L-DOPA in its ability to control motor symptoms [320]. At the same time, apomorphine does not affect pain symptoms in patients with PD, which indicates other monoamine systems are involved in the pathogenesis [321]. MAOs exert a significant impact on the course of PD, as they are involved in the metabolism of DA. MAOs inhibitors, such as rasagiline or selegiline, inhibit the metabolism of DA and L-DOPA, prolonging their action. By reducing MAO-B activity, they protect neurons from oxidative damage [322]. A similar effect is exerted by catechol O-methyltransferase (COMT) inhibitors, such as entacapone [36,323]. 

### 10.2. Non-Traditional Pharmaceuticals for Prevention and Treatment PD

Amantadine was originally discovered as an anti-viral to treat influenza in the 1950s. In the late 1960s, it was discovered to be useful in treating tremors and dyskinesia associated with Parkinson’s disease and began to be widely used for this purpose. Today amantadine is prescribed for some chronic neurodegenerative and neurocognitive diseases. The mechanism of action of amantadine is largely unknown. Amantadine keratopathy is a term used to describe corneal edema and subsequent decrease in visual acuity that is assumed to be caused by the drug. Corneal edema typically resolves with discontinuation of the drug, although cases requiring corneal transplants have been reported [324].

Using an unbiased screen targeting endogenous gene expression, the β2-adrenoreceptors (β2AR) were discovered to be regulators of the αSyn gene [325]. Salbutamol, a β2AR agonist, reduced the risk of developing PD and protected PD patient-derived cells and model mice. On the other hand, a β2AR antagonist correlated with increased risk.

Ambroxol, which was initially used to treat airway mucus hypersecretion and hyaline membrane disease in infants and was then recognized as a pH-dependent, mixed-type inhibitor of β-glucocerebrosidase 1 (GCase 1, a lysosomal hydrolase), acts as a chaperone for GCase1 and enhances lysosomal function and autophagy. Clinical trials of ambroxol were described in PD and PD dementia [326]. These studies might also provide new and alternative strategies to treat PD.

Ursodesoxy cholic acid (UDCA) or tauro ursodesoxy cholic acid (TUDCA) treatment improved motor performance, ameliorated mitochondrial dysfunction and neuroinflammation, and prevented the decline of striatal dopamine content in various PD models [203]. TUDCA is an anti-apoptotic agent that upregulates mitophagy and can upregulate the expression of PINK1 and parkin in SH-SY5Y cells to accelerate the clearance of damaged mitochondria, promoting the survival of damaged neurons [327]. Moreover, in the PD model of rats, UDCA treatment rescued the DA content in the striatum and relieved the motor symptoms by downregulating the expression of Bax, maintaining the integrity of the mitochondrial membrane. This effect was accompanied by a decrease in the expression of the pro-apoptotic pathway including caspase-9, caspase-3, and caspase-8 [328]. TUDCA and UDCA maintain mitochondrial function to reduce the damage of dopaminergic neurons by accelerating the clearance of damaged mitochondria and reducing the expression of pro-apoptotic pathways [203,329].

Additionally, the mitochondrial damage caused by PARPs was rescued by adding NAM to the diet [330]. NAM is the amide form of vitamin B3 and a metabolite of the NAD+ salvage pathway, its supplementation maintains mitochondrial function by increasing NAD levels to enhance the metabolism of dopaminergic neurons, thereby improving PD pathology [203].

Biperiden is a drug used in Parkinson’s disease treatment and it serves also as an antiseizure compound in OPs poisoning. It acts as an antagonist of muscarinic receptors and appears to be a very weak inhibitor, though it can serve as a lead structure in pharmacological research [331].

### 10.3. Targeting Autophagy in PD

Autophagy is a potential target for PD treatment since it initiates the clearance of protein aggregates and inhibits apoptosis [332]. Molecular chaperones have crucial roles in inhibiting the aggregation of misfolded proteins. Small heat shock proteins (sHsps) are key elements of the proteostasis network, playing a critical role in inhibiting the aggregation of misfolded proteins. sHsp Hsp27 (HSPB1) has been shown to bind along the surface of αSyn fibrils and reduce their hydrophobicity, inhibit growth, prevent elongation, and thereby inhibit the cytotoxicity of αSyn fibrils [333].

Autolysosome pathway homeostasis (ALP) is closely associated with PD, and disruption of autophagy can cause neuronal death and thus accelerate the progression of PD. The use of autophagy to solve pharmacological problems with the help of small molecular weight compounds is attracting increasing attention. The main targets associated with autophagy are AMPK, mTORC1, ULK1, IMPase, LRRK2, beclin-1, TFEB, GCase, ERRα, C-Abelson, and related small molecule compounds. Autophagy modulators include natural compounds and synthesized pharmaceuticals that alter AMPK activity. The former includes resveratrol and caffeine [334,335], the latter include metformin [336], A769662 and GSK621 [337], Rosuvastatin [184], FCPR16 [338], and Temozolomide [339]. Of the natural compounds that act mainly on mTORC1, we note rapamycin and its analogs CCI-779 and AP23573 [340,341], Corynoxine [342], and Loganin [343]. Among the artificial mTORC1 activators, we note PI-103, which increases the clearance of αSyn in LUHMES (Lund human mesencephalic cells) [339]. 

Inositol monophosphatase (IMPase) inhibitors are chemical compounds under investigation for use in PD; the action of which is associated primarily with the activation of autophagy: sodium valproate [344], Carbamazepine [344], and L-690.330 [339]. Leucine-rich repeat kinase 2 (LRRK2) inhibitors are also man-made chemical compounds, such as LRRK2-IN-1 [345], GNE-7915 [346], PF-06447475 [347], DNL151 and DNL201 [348]. Natural inhibitors of Beclin-1-Isorhynchophylline [349], Corynoxine B [350], and Glycyrrhizic acid [351]; KYP-2047 inhibitor was synthesized [352]. Curcumin and trehalose are natural modulators of TFEB-mediated autophagy [353,354,355]. 

Compounds promising for the treatment of PD that increase the amount and/or activity of lysosomal glucocerebrosidase (GCase) have been synthesized, and include Ambroxol, Isofagomine, and NCGC607 [356,357,358,359]. Promising inhibitors of Abl Kinase (c-ABL) have also been synthesized, e.g., PD180970, Imatinib, and Nilotinib [360,361,362,363].

### 10.4. Care for Vascular Endothelium in PD

The problem of prevention of neurodegenerative diseases, such as PD, and therapeutic care for patients with clinical signs of these diseases should be carried out taking into account the endothelial component of pathogenesis [223]. In this regard, it should be noted that at present special attention is paid to the peculiarities of lifestyle, in particular, indicators of the quality of the diet, food components and its calorie content. A high-calorie, high-fat diet that can induce type 2 diabetes (DMT2) in mice does not affect the extent of dopaminergic neuronal damage in a mouse model of PD but exacerbates the clinical manifestations of PD. It is important to note that such a diet leads to a significant depletion in pericytes and a decrease in the interaction of microglia with vessels, which indicates an aggravation of vascular pathology [237]. A relationship has been established between maternal obesity and the development of neurodegenerative diseases in children [364]; a positive effect of a ketogenic diet and intermittent fasting as preventive and even therapeutic factors in neurodegenerative diseases has been reported [365,366,367]. This is especially important for assessing the state of the endothelium and correcting vascular diseases. There is a close relationship between nutritional structure and the expression of endothelial markers [222,255]. Conditionally healthy foods or meals, in particular, fruits and vegetables, consumed regularly, have a positive effect on the functional state of the endothelium, which can be assessed by the level of its markers, such as sICAM-1, sVCAM-1, E-selectin, and some others. Recently, there has been a reassessment of the role of albumin, and its oxidized and glycated derivatives in the development of cardiovascular and neurodegenerative diseases [292]. A western diet (predominantly meat, sweets, refined foods and fried foods) correlates with markers of inflammation, atherogenesis, and neurodegeneration [368]. New concepts, research methods, and new ways of targeting cells and the body as a whole give us confidence in developing more natural approaches to solving problems of cerebrovascular health, and mental and neurodegenerative diseases in humans.

### 10.5. Use of Nutraceuticals in PD 

LB accumulates in special cellular compartments of special brain areas for many years before motor dysfunction manifests, suggesting that disease-modifying therapy should start earlier during the premotor stage. Long-termed regulation of lifestyle, diet and supplement of nutraceuticals may be possible ways for the disease-modification. Diet can reduce the incidence of PD and phytochemicals, major bioactive ingredients of herbs and plant food, modulate multiple pathogenic factors and exert neuroprotective effects in preclinical studies [369]. Growing evidence indicates that the herbs used in traditional medicines contain neuroprotective compounds such as resveratrol, curcumin or ginsenoside, green tea polyphenols or catechins, triptolide, etc. [370,371,372]. In experiments predominantly in vitro and in animal models, it has been shown that they promote neuronal survival and neurite growth and facilitate functional recovery by invoking distinct mechanisms that are related to their roles as antioxidants, dopamine transporter inhibitors, monoamine oxidase inhibitors, free radical scavengers, chelators of harmful metal ions, modulating cell survival genes and signaling, anti-apoptosis activity, and even improving brain blood circulation. Garlic and allicin, cabbage and isothiocyanates, chickpea seed and its O-methylated isoflavones biochanin A and formononetin, cinnamon, and cinnamaldehyde, saffron and its crocin, crocetin, and safranal, black cumin and its thymoquinone, black pepper and piperine, pistachio and genistein and daidzein, and resveratrol are among the most effective dietary items against PD [334]. They act by attenuating neurotoxin-induced memory loss and behavioral impairment, oxidative stress, and dopaminergic cell death, thus alleviating PD progression. People with very high levels of the antioxidants lutein, zeaxanthin, and beta-cryptoxanthin (carotenoids) in their blood have a much lower risk of developing dementia in old age [373]. Resveratrol is a polyphenolic compound found in grapes, it is able to cross the BBB and is water soluble. Numerous pharmacological functions include anti-inflammatory, anti-apoptotic, antioxidant, and anti-cancer activities, as well as preventing the development of PD [374,375]. Curcumin has shown therapeutic potential in neurodegenerative diseases, including PD. It is a natural polyphenol found in spicy turmeric, known for several biological and medicinal effects, such as anti-inflammatory, antioxidant, anti-proliferative, etc. It has been shown to help prevent αSyn aggregation and fibrillation [376]. Curcumin glucoside prevents aggregation and increases the solubility of αSyn [377]. Blueberries are high in polyphenols and have stronger antioxidant properties than most fruits and vegetables. In a unilateral intrastriatal 6-hydroxydopamine (6-OHDA) rat model of PD, blueberry consumption has been reported to slow age-related functional and physiological deficits, preventing the development of PD [378]. 

Accumulated data indicate that sulfane sulfur performs important functions in cells, and it is modulated primarily by organosulfur compounds (OSC) [379]. The wide variety of effects suggests that its functions are general and not specific to any tissue or any process. Previously, the idea of “orthogonal control” was substantiated, the meaning of which is the existence of redox regulatory mechanisms as more global, in contrast to the mechanisms of “redox signaling”, which include the transmission of discrete activating or inactivating signals [380]. Within this paradigm, regular use of nutraceuticals takes on new meaning, which could serve as a kind of “pro-electrophilic drugs” that become electrophilic in response to oxidation and then activate the Keap1/Nrf2/ARE transcription pathway to synthesize endogenous antioxidant “phase 2” enzymes [381]. These substances are also regarded as pathology-activated therapeutics that are converted to their active form by oxidative stress, which they then have to combat [369,381]. This “electrophilic counterattack” could be organized by the administration of OSC-containing nutraceuticals, which exert their effects not only through their original properties quickly reacting with or generating ROS, but rather through mobilization and/or coordination of necessary “weapons” in definite areas of the “battlefield” [379]. 

### 10.6. Targeting Gut Microflora

Intestinal bacteria can cause motor deficits, microglia activation and αSyn pathology; therefore, changes in the human microbiome may increase the risk of PD [203,204]. The mammalian microbiome is complex and composed of 300 to 1000 bacterial species with a total number that exceeds that of host cells, and not all of the species are well studied, many of them are not cultured and therefore not studied at all, which is a serious obstacle to understanding their biological roles [382]. They are affected by various endogenous and exogenous factors. The recovery and maintenance of gut microbiota may represent a therapeutic option for diseases related to dysbiosis. Targeting the gut microbiota using probiotics, antibiotics, and fecal microbial flora transplantation (FMT) may restore the composition of the gut microbiota, replenish beneficial metabolites, and reduce harmful metabolites to address the pathophysiology and mitigate the symptoms of PD.

Probiotics are living microbial preparations [383]. In mice, the administration of *Lactobacillus rhamnosus* increased the expression of GABA in the brain and reduced anxiety and depression-related behaviors [384]. A mixed probiotics preparation (*Bifidobacterium lactis*, *Lactobacillus acidophilus*, *Lactobacillus paracasei*, and *Lactobacillus plantarum*) raised the level of SCFA in in vitro experiments in the human colon [385]. In patients with mental illness, some psychobiotics defined as live bacteria are considered to relieve mental symptoms by promoting the synthesis of endogenous neurotransmitters, such as GABA, catecholamines, and 5-HT [386]. Several studies have revealed the benefits of probiotics in patients with PD, including the alleviation of constipation and motor symptoms [387]. Nevertheless, to date, there is no reliable clinical data to prove the possible influence of probiotic treatment on motor symptoms or PD progression.

Prebiotics are soluble dietary fibers that stimulate the growth of gut commensal microbiota to combat disease and maintain health. In mice, long-term use of fructooligosaccharides (FOS) and galactooligosaccharides (GOS) significantly improved anxiety and depression-related behaviors by increasing SCFA-producing bacteria [388]. Combined use of prebiotics and probiotics alleviated mitochondrial dysfunction in the brain of mice fed a high-fat diet [389], indicating that increasing beneficial bacteria in the intestine has the potential to attenuate CNS disease. The consumption of fermented milk containing prebiotic fiber improved constipation in PD patients [390]. Prebiotics may improve the pathology of PD by stimulating the colonization of beneficial microorganisms in the intestine and promoting the secretion of SCFA [203].

Antibiotics are well known to kill microorganisms, but they can also inhibit the accumulation of abnormal proteins and improve mitochondrial function, which might be beneficial for the treatment of neurodegenerative disease [391,392,393]. On the other hand, exposure to certain oral antibiotics may increase the risk of PD possibly due to the long-term effects of antibiotics on the composition of the human gut microbiota [394].

FMT is a therapeutic strategy in which feces from healthy donors are delivered to patients to achieve a therapeutic effect by restoring a stable gut microbial environment [395]. In a mouse model of PD induced by MPTP, FMT altered the gut microbiota imbalance, resulting in increased levels of DA and 5-HT in the striatum [197]. Patients with multiple sclerosis, myoclonus dystonia, autism, depression, and PD may also benefit from FMT, and clinical trials are currently underway to treat PD with FMT [396]. Although FMT is an attractive technique, many questions regarding its safety and effectiveness remain to be answered before it can be applied in PD treatment.

### 10.7. Physiotherapy and Exercise in PD

Although surgery and medications have positive effects on PD, they have little effect on postural instability and gait problems, and also have many side effects [397]. Physical, occupational, and speech therapies provide non-drug alternatives that can be used in adjunct with medications, or separately for those who prefer more natural approaches [36,398]. The future of PD treatment is promising for patient-specific care that is often more effective and with minimal side effects

Physiotherapy is a simple yet effective treatment for PD. Deep brain stimulation (DBS) has been developed as an alternative to ablative operations in the globus pallidus, subthalamic nucleus and thalamus [308,399,400]. It is a recognized treatment for advanced stages of PD, improving motor symptoms in the short and long term. Physical exercise is now often used as an important part of the treatment of PD [401,402,403]. Moderate physical activity has a positive effect on both endothelial markers and cognitive functions in the elderly [404]. An increasing number of studies have examined the impact of non-pharmacological/non-surgical supportive “activating” therapies, which include physical exercise, dance classes, and speech therapy [405]. Exercise improved the level of physical and mental functioning and quality of life of patients with PD [406,407,408,409,410]. Treadmill training, dance and yoga can significantly improve gait parameters, other motor performance and overall quality of life [411,412,413]. Thermal aquatic exercise may represent a promising rehabilitation tool to prevent the impact of motor symptoms on the daily-life activities of people with PD [414].

In animal models of PD, exercise improves the specific motor symptoms of PD and reduces the loss of dopamine neurons [415]. In model experiments with rotenone-induced PD in rats, it was shown that treadmill running inhibited the formation of LB in the nigrostriatum [49]. Moreover, the incubation of primary neurons and astrocytes with lactate or pyruvate activated mitophagy and autophagy after MPP + treatment. Particularly, acidification of the cytosol caused by pyruvate or lactate led to the restoration of mitochondrial function and protection of these cells from apoptotic and necrotic death [416].

### 10.8. Experimental Stem Cell Therapy

Mesenchymal stem cells (MSCs) are regarded to be very promising in treating PD, as they can differentiate into dopaminergic neurons and produce neurotrophic substances. The precise process by which stem cells repair neuronal injury is unknown, and MSC-derived exosomes are suggested to be responsible for a significant portion of such effects [417,418]. Moreover, using three transcription factors (NEUROD1, LMX1A, and ASCL1), miR218 and collectively designated NeAL218, DA neurons are generated by directly reprogramming human astrocytes in vitro and mouse astrocytes in vivo [419].

### 10.9. Immunotherapies

One more approach to cope with PD, which engendered much hope, has been targeting the pathologically aggregated form of αSyn [420]. To this end, extracellular disulfide bond-free synthetic nanobody libraries in yeast were designed [421]. Following selection, a nanobody, PFFNB2, was identified that could not inhibit the aggregation of αSyn monomer, though it can specifically recognize and significantly dissociate αSyn fibrils. A series of humanized monoclonal antibodies BIIB054 (cinpanemab), MEDI1341, AFFITOPE, and PRX002 (prasinezumab) were under clinical development [422]. However, despite the huge financial costs, the results of these developments were rather discouraging. In patients with early PD, the effects of cinpanemab on clinical measures of disease progression did not differ from those of a placebo over a 52-week period [423]. Additionally, prasinezumab therapy had no meaningful effect on global or imaging measures of Parkinson’s disease progression as compared with placebo and was associated with infusion reactions [424].

### 10.10. Epigenetic Drugs

Any drug intervention is believed to reverse the epigenetic mechanisms to serve as a regulator in neuronal diseases. In recent years, epigenetic drugs have shown unexpected therapeutic effects on neurological diseases, leading to the study of the epigenetic mechanisms in neurodegenerative diseases [425]. For example, in the MPTP-induced PD model in mice, scorpion extract consistently attenuated the decrease of tyrosine hydroxylase-positive neural cells in the SN and striatum of mice [426]. The epigenetic targets in its therapeutic mechanism were 13,479 differentially expressed genes, mainly in the promoter and coding regions, which were identified in association with the anti-PD effect of the scorpion extract. Folate, vitamin B6, vitamin B12, and S-adenosylmethionine are vital cofactors involved in DNA methylation modification; 5-azacytidine is the most widely studied DNMT inhibitor, and dietary polyphenols are DNMT inhibitors in vitro [427]. 

Until now many reports on drug discovery focused on inhibiting HDAC and DNMTs to reverse the epigenetic changes; unfortunately, they lack targeted delivery and sometimes cause a cytotoxic effect on neuronal cells [428]. 

To better understand the data presented in Section 10, the reader can refer to Table 2, in which medicines, substances and various methods are listed that can slow down or prevent the development of PD.

## 11. Conclusions

**Figure 3:**
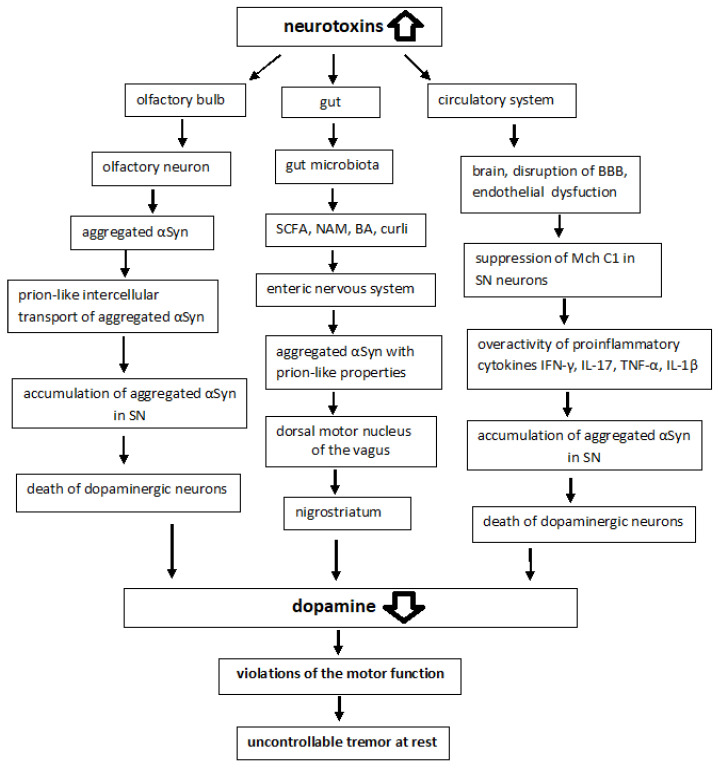
Sporadic forms of PD are caused by environmental factors, such as neurotoxins, vascular risk factors, physical inactivity, etc., and characterized by abnormal accumulations of αSyn within neuronal cell as Lewy bodies, which first appear in the olfactory bulb, the gut nervous system, and the dorsal motor nucleus of the vagus located in the medulla oblongata, then in the locus coeruleus of the pons, the raphe nucleus in the pontine midbrain, SNpc in the midbrain, and finally Lewy bodies are found in the temporal cortex, limbic region, and cerebral cortex. PD is characterized not only by the abnormal accumulations and aggregation of αSyn within neuronal cell bodies and neuritis, but also by intercellular transport of aggregated αSyn. MCh are the primary target of external influence in sporadic forms of PD.

In accordance with the scientific approach, in order to understand the intricacies of intermolecular and intercellular relationships underlying the pathogenesis of any disease including PD, it is necessary to move in two directions: the medical and biological examination of patients at different stages of the disease and the development/use of adequate experimental models using animal or cellular cultures. Adherence to the principles of translational medicine involves extensive cooperation between basic researchers, clinicians, and industry to finally generate numerous new targeted compounds with enhanced efficacy and decreased toxicity. The failure to translate insights gained from animal or in vitro models into humans is not always due to flaws of the models *per se*. For example, many of these studies lacked detailed causal experimentation and did not exclude other variable factors. An increase in proper causal experimental design using robust engineered models that recapitulate the complexity of an entire nervous system, including a full complement of neuronal circuits, glial complexity, and immunologic and vascular components will provide valuable insight into how mitochondria impact disease pathogenesis [111]. The Braak hypothesis proposes that in PD a pathologic agent may penetrate the nervous system via the olfactory bulb, gut, or both and spreads throughout the nervous system, hitting mitochondria along the way. In this regard, of the most challenging questions that remain to be answered, one seems to be central [111]: Do metabolic defects and mitochondrial dysfunction cause neurodegeneration, or does neuronal dysfunction result in metabolic defects and mitochondrial dysfunction? The second important question is tightly bound with the first one: Do metabolic defects cause microvascular damage, or does microvascular damage result in metabolic defects and mitochondrial dysfunction? Metabolic dysfunction often affects microvascular ECs through albumin modifications, along with oxidative stress and inflammation, and promotes the damage of endothelial integrity followed by albumin penetration to the brain parenchyma [292]. The problem of prevention of neurodegenerative diseases such as PD, and therapeutic care for patients with clinical signs of these diseases should be carried out taking into account the “endothelial” constituent of pathogenesis [223,255]. In this regard, we suggest that the Braak dual hit hypothesis should be modified to the ‘triple hit hypothesis’; the pathogenesis resembles a ‘three lane highway’ Figure 3. 

αSyn accumulates in enteric neurons prior to motor symptoms and can spread from the gut to the brain via microtubules in a “prion-like” manner. Prions can cause CNS disease through multiple transmission routes, including intracerebral, intraperitoneal, and intravenous. The gut microbiota converts substrates into various metabolites including short-chain fatty acids (SCFA), niacinamide (NAM), bile acids (BA), curli and neurotransmitters. Enteroendocrine cells of the intestinal epithelium can contribute to the spread of pathological αSyn. Additionally, disturbances in intestinal permeability and systemic exposure to bacterial antigens induce the expression of inflammatory cytokines, which disrupt the integrity of the BBB and lead to the death of dopaminergic neurons.

Inflammation caused by aggregated αSyn causes EC activation, which manifests itself in EC contraction, increased paracellular permeability, and generation of pro-inflammatory cytokines and chemokines. These factors cause an increase in local blood flow, BBB dysfunction, extravasation of protein-rich exudate, and the recruitment and activation of circulating leukocytes, which further contribute to neuroinflammation. Erythrocyte-derived exosomes containing αSyn induce microglial activation in PD patients. Lymphocytes are also involved in the pathogenesis of PD, mainly due to the infiltration of T cells with increased expression of LFA1, a receptor for ICAM-1 on endothelial cells. 

In this regard, it should be mentioned that while dopamine replacement therapies improve symptoms, they do not modify the course of the disease. At present, in addition to traditional and non-traditional therapies, particular attention is paid to lifestyle features, in particular, indicators of the quality of the diet, nutritional components and its calorie content, that is, factors that largely affect the state of the vascular wall and blood vessels in general. Thus, a link has been established between maternal obesity and the development of neurodegenerative diseases in a child [364]. The positive effect of the ketogenic diet has been established; intermittent fasting is preventive and even therapeutic in neurodegenerative diseases [365,366,367]. This is especially important for assessing the state of the endothelium and correcting vascular diseases. There is a close relationship between nutritional structure and the expression of endothelial markers. Conditionally healthy foods or meals, in particular, fruits and vegetables, consumed regularly, have a positive effect on the functional state of the endothelium, which can be assessed by the level of its markers, such as sICAM-1, sVCAM-1, E-selectin, and some others. Recently, the role of albumin has been revisited, with an accent of its oxidized and glycated derivatives in the development of cardiovascular and neurodegenerative diseases [292]. 

More and more evidence has emerged that that PD is not a single entity but instead reflects multiple diseases, in which different combinations of environmental, genetic and potential comorbid factors interact to direct individual disease trajectories. Moreover, an increasing body of recent research implicates peripheral tissues and non-neuronal cell types in the development of PD. These observations are consistent with the hypothesis that the initial causative changes for PD development need not occur in the central nervous system [429]. The clear consequence of there being distinct diseases that collectively form PD is that there is no single biomarker or treatment for PD development or progression. To enable informative patient stratification, an important conclusion for physicians and neurologists is that diagnosis should shift away from the clinical definitions toward biologically defined diseases that collectively form PD. A personalized or N-of-one type clinical designs offer an unbiased and agnostic approach to re-defining PD in terms of a group of many individual diseases [429].

New concepts, research methods, and new ways of targeting cells and the body as a whole give us confidence in developing more natural approaches to solving problems of cerebrovascular health, mental and neurodegenerative diseases in humans. Pharmacological, surgical, and therapeutic treatments have allowed physicians to treat not only the main motor symptoms of PD but target patient-specific problems as they arise. To this end, long-term and large trials are needed to evaluate the differences in mortality, adverse events, and morbidity of PD after the changes in lifestyle and nutraceutical therapy. It is particularly important from the point of view of seeking new biomarkers for various pathological states. Machine learning methods should be more widely applied to the diagnosis of PD and potential biomarkers for assisting clinical decision-making [430]. These approaches have the potential to provide clinicians with additional tools to screen, detect or diagnose PD, and to monitor the effectiveness of therapy.

## Figures and Tables

**Table 1:** Endothelial cell functions in relation to pathogenesis of PD.

Physiological Functions	Regulatory Factors	Involvement in Pathogenesis of PD
Inflammation	IL-1β, IL-6, TNF-α, MMP-9, MCP-1, Complement activation, Oxidative stress	[224,225,226,227,228,229]
Leukocyte trafficking	ICAM1, VCAM, E-selectin	[196,228,230]
Haemostasis and coagulation	Anticoagulant factors: TM, tPA, PGI_2_ Procoagulant factors: vWF, TXA_2_, PAI1	[143,231,232,233,234,235,236]
Control of VSMC and pericytes, Vascular tone	Microparticles, Jagged-1/NOTCH-1, miR-126, NO	[228,237,238,239,240,241,242,243]
Vascular permeability, Angiogenesis and Metabolism	VEGF/VEGFR, HIF activation, Nogo-B, Glutamate metabolism, Jagged-1/NOTCH-1	[227,230,238,244,245,246,247,248,249,250]

**Table 2:** The substances or interventions for the treatment of PD, their targets or mechanisms of action.

##	Substances or the Type of Intervention	The Target or Mechanism of Action	References
1	pallidotomy, thalamotomy, subthalamotomy	reducing excessive inhibition from the globus pallidus and SN	[36,308]
2	L-DOPA or levodopa	substitution for DA loss in the striatum	[310]
3	pergolide, pramipexole dihydrochloride, ropinirole hydrochloride, rotigotine, apomorphine hydrochloride	DA-like agonists that bind to dopaminergic postsynaptic receptors	[319,320]
4	rasagiline, selegiline,	MAO inhibitors, prolongation of DA and L-DOPA action	[321,322]
5	entacapone	COMT inhibitor	[36,323]
6	salbutamol	β2AR agonist, regulators of the αSyn gene	[325]
7	UDCA, TUDCA	anti-apoptotic agents, upregulates mitophagy and the expression of PINK1 and parkin	[203,327]
8	flavonoids and carotinoids	reduction of excessive ROS production and oxidative stress	[314,315]
9	NAM	maintains mitochondrial function by increasing NAD levels in dopaminergic neurons	[203,330]
10	biperiden	antagonist of muscarinic receptor	[331]
11	Hsp27 (HSPB1)	binding of αSyn fibrils and inhibition their cytotoxicity	[333]
12	resveratrol, caffeine, metformin, A769662, GSK621, rosuvastatin, FCPR16, temozolomide	autophagy modulators by activating AMPK	[184,334,335,336,337,338,339]
13	rapamycin, its analogs CCI-779 and AP23573, corynoxine, loganin, PI-103	autophagy modulators by activating mTORC1	[339,340,341,342,343]
14	sodium valproate, carbamazepine, L-690.330	autophagy modulators by inhibiting IMPase	[339,344]
15	LRRK2-IN-1, GNE-7915, PF-06447475, DNL151, DNL201	autophagy modulators by inhibiting LRRK2	[345,346,347,348]
16	isorhynchophylline, corynoxine B, glycyrrhizic acid, KYP-2047	autophagy modulators by inhibiting Beclin-1	[349,350,351,352]
17	ambroxol, isofagomine, NCGC607	autophagy modulators by inhibiting GCase 1	[326,356,357,358,359]
18	fruits and vegetables, nutraceuticals and phytochemicals	antioxidant, anti-inflammatory and anti-apoptotic activities; improvement of the functional state of endothelium and brain blood circulation; modulation of the cell survival genes	[292,334,369,370,371,372,373,374,375,376,377,378]
19	probiotics, prebiotics, antibiotics, FMT	restore the composition of the gut microbiota, replenish beneficial metabolites, and reduce harmful metabolites	[203,204,383,384,385,386,387,388,389,390]
20	deep brain stimulation	an alternative to ablative operations in the globus pallidus, subthalamic nucleus and thalamus	[308,399,400]
21	moderate physical activity	positively affects endothelial cells and cognitive functions, gait parameters and other motor performance	[404,405,408,409,410]
22	mesenchymal stem cells (MSCs)	differentiate into dopaminergic neurons and produce neurotrophic substances	[417,418]
23	monoclonal antibodies and nanobodies	targeting the aggregated αSyn	[421,422,423,424]
24	folate, vitamin B6, vitamin B12, and S-adenosylmethionine, 5-azacytidine, dietary polyphenols, scorpion extract	drugs and substances affecting epigenetic modifications—DNA methylation and histone acetylation	[426,427,428]

## Data Availability

Not applicable.

## Abbreviations

ACOX	acyl-CoA oxidase
AD	Alzheimer disease
ALS	amyotrophic lateral sclerosis
AP	action potential
AP-1	activator protein 1
ALP	autophagy-lysosome or autolysosome pathway
AQP4	aquaporin 4
αSyn	α-synuclein
BA	bile acids
BAM	border-associated macrophages
BBB	blood-brain barrier
BG-PVS	perivascular space of the basal ganglia
c-ABL	Abl kinase
CAA	cerebral amyloid angiopathy
CNS	central nervous system
COMT	catechol O-methyltransferase
CPF	chlorpyrifos
DA	dopamine
DAO	D-amino-acid oxidase
DAMP	damage-associated molecular patterns
DATs	dopamine transporters
DBS	deep brain stimulation
DDH1 or AKR1C1	dihydrodiol dehydrogenase
DDO	D-aspartate oxidase
DLB	dementia with Lewy bodies
DMT2	type 2 diabetes
DNMTs	DNA methyltransferases
DMV	dorsal motor nucleus of the vagus
DOPAL	3,4-dihydroxyphenylacetaldehyde
DSCs	disease-specific single-nucleotide changes
DTI-ALPS	diffusion tensor image analysis along the perivascular space
EECs	enteroendocrine cells
EET	epoxyeicosatrienoic acids
EC	endothelial cells
ENS	enteric nervous system
ER	endoplasmic reticulum
ETC	electron transport chain
EVs	erythrocyte vesicles
FAO	fatty acid β-oxidation
FHC	ferritin heavy chain
FMT	fecal microbial flora transplantation
FOS	fructo-oligosaccharides
GABA	γ-aminobutyric acid
GBA1	lysosomal glucocerebrosidase
GCase 1	β-glucocerebrosidase 1
GI	gastrointestinal tract
GOS	galacto-oligosaccharides
GPx	glutathione peroxidase
GST-Pi	glutathione-S-transferase Pi
GWAS	genome-wide association study
HAO	L-α-hydroxyacid oxidase
HDAC	histone deacetylase
5-HT	5-hydroxytryptamine, serotonin
ICAM-1	Inter-Cellular Adhesion Molecule 1
ICDH	isocitrate dehydrogenase
IL-1β	interleukin-1β
iLBD	incidental LB disease
IMM	inner mitochondrial membrane
IMS	intermediate syndrome
InsP3R	inositol 1,4,5-trisphosphate receptors
Kir6	ATP-sensitive K^+^ channels
LB	Lewy bodies
LC	locus coeruleus
LRRK2	leucine-rich repeat kinase 2
LPS	lipopolysaccharide
LRRK2	leucine-rich repeat kinase 2
LTCCs	L-type calcium channels
LUHMES	Lund human mesencephalic cells
MAM	mitochondria-associated membranes
MAO	monoamine oxidase
MAO-B	monoamine oxidase B
MCh	mitochondria
MCP-1	monocyte chemotactic protein-1
miRNA	microRNA
MMP-9	matrix metalloproteinase-9
mPTP	mitochondrial permeability transition pore
MPTP	1-methyl-4-phenyl-1,2,3,6-tetrahydropyridine
MPP+	1-methyl-4-phenylpyridinium
MT3	metallothionein-3
NAC	non-amyloid-β component
NAM	niacinamide
NC	nucleus caudatus, caudate nucleus
ncRNA	non-coding RNA
NO	nitric oxide
Nogo-B	Neurite outgrowth inhibitor-B
NOX	NADPH oxidases
3-NPA	3-nitropropionic acid
nt	nucleotides
NVU	neurovascular unit
OGDH	oxoglutarate dehydrogenase
6-OHDA	6-hydroxydopamine
OMM	outer mitochondrial membrane
OPs	organophosphates
OPIDP	organophosphate-induced delayed neuropathy
OSC	organosulfur compounds
PAI-1	plasminogen activator inhibitor-1
PAOX	polyamine oxidase
PD	Parkinson’s disease
PDD	Parkinson’s disease dementia
PDH	pyruvate dehydrogenase
PFFs	pre-formed fibrils
PINK1	phosphatase and tensin homologue (PTEN)-induced kinase 1
PIPOX	L-pipecolic acid oxidase
PKA	protein kinase A
PMCA	plasma membrane Ca^2+^-ATPase
PNS	peripheral nervous system
PON-1	paraoxonase-1
PP2A	protein phosphatase 2A
PrP	prion proteins
PVM	perivascular macrophages
ROS	reactive oxygen species
SCFA	short-chain fatty acids
sHsps	small heat shock proteins
SN	substantia nigra
SNpc	substantia nigra pars compacta
SOD	superoxide dismutase
TJ	tight junction
TM	thrombomodulin
TNF-α	tumor necrosis factor alpha
TOM	translocase of the outer membrane
TOM-CC	TOM core complex
tPA	tissue plasminogen activator
TUDCA	tauro ursodesoxy cholic acid
UDCA	ursodesoxy cholic acid
VCAM-1	vascular cell adhesion molecule 1
VDAC	voltage-dependent anion channel
VEGFR	vascular endothelial growth factor receptor
VMAT2	vesicular monoamine transporter 2
VPS35	vacuolar protein sorting ortholog 35, involved in autophagy
VTA	ventral tegmental area
vWF	von Willebrand factor
WPB	Weibel-Palade bodies
XO	xanthine oxidase
ZO-1	zonula occludens-1
